# Synthesis of 2-((2-(Benzo[d]oxazol-2-yl)-2*H*-imidazol-4-yl)amino)-phenols from 2-((5*H*-1,2,3-Dithiazol-5-ylidene)amino)phenols through Unprecedented Formation of Imidazole Ring from Two Methanimino Groups

**DOI:** 10.3390/molecules25173768

**Published:** 2020-08-19

**Authors:** Ilia V. Baranovsky, Lidia S. Konstantinova, Mikhail A. Tolmachev, Vadim V. Popov, Konstantin A. Lyssenko, Oleg A. Rakitin

**Affiliations:** 1 N. D. Zelinsky Institute of Organic Chemistry, Russian Academy of Sciences, 119991 Moscow, Russia; ilay679@rambler.ru (I.V.B.); konstantinova_ls@mail.ru (L.S.K.); mtolmachev4@gmail.com (M.A.T.); 2 Nanotechnology Education and Research Center, South Ural State University, 454080 Chelyabinsk, Russia; popov.ioc@gmail.com; 3 Department of Chemistry, M. V. Lomonosov Moscow State University, Leninskiye Gory, 1, 119991 Moscow, Russia; kostya@ineos.ac.ru; 4 G. V. Plekhanov Russian University of Economics, 36 Stremyanny Per., 117997 Moscow, Russia

**Keywords:** sulfur-nitrogen heterocycles, 5-Arylimino-1,2,3-dithiazoles, four substituted 2*H*-imidazol-4-amines, X-ray analysis, thermolysis

## Abstract

A new synthetic pathway to four substituted imidazoles from readily available 2-((4-aryl(thienyl)-5*H*-1,2,3-dithiazol-5-ylidene)amino)phenols has been developed. Benzo[*d*]oxazol-2-yl(aryl(thienyl))methanimines were proved as key intermediates in their synthesis. The formation of an imidazole ring from two methanimine derivatives likely includes the opening of one benzoxazole ring followed by ring closure by intermolecular nucleophilic attack of the *N*-methanimine atom to a carbon atom of another methanimine.

## 1. Introduction

**Scheme 1:**
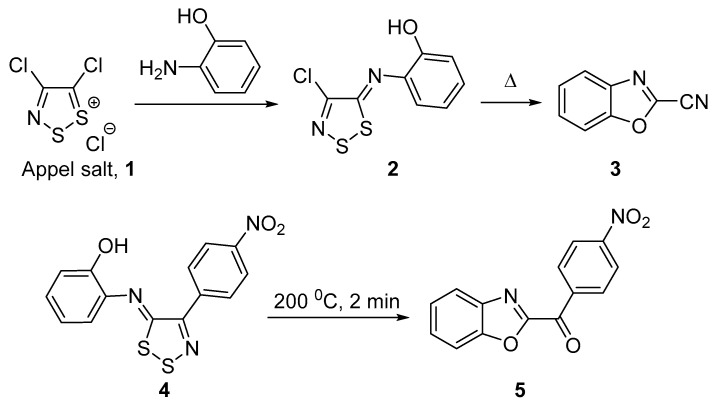
Synthesis of 5-arylimino-1,2,3-dithiazoles **2** and **4** and subsequent formation of benzo[*d*]oxazole derivatives **3** and **5**.

1,2,3-Dithiazoles are one of the most investigated groups of five membered sulfur–nitrogen heterocycles [1,2,3]. In addition to the utility of these heterocyclic compounds as potent biologically active compounds [4,5,6,7,8], they are efficient precursors for functional materials applied in electronics and spintronics [9,10,11,12,13,14]. The chemistry of monocyclic 1,2,3-dithiazoles has attracted considerable attention through recent decades [1,2,3] due to the easy availability of 4,5-dichloro-1,2,3-dithiazolium chloride (Appel’s salt, **1**) [15,16]. 4-Chlorosubtituted monocyclic 1,2,3-dithiazoles [1,2,3,17,18,19,20] were thoroughly investigated. The most interesting and valuable parts of this reactivity are various rearrangements of 4-chloro-1,2,3-dithiazoles, especially 5-arylimino derivatives (i.e., **2**), which showed a great potential for the synthesis of multiple heterocycles, such as 1,2,4-thiadiazoles [21], isothiazoles [22], benzoxazines and benzothiazines [23], benzimidazoles [24], quinazolones [25], benzothiazoles [26] and benzoxazoles (**3**, see Scheme 1) [26,27,28], and many others [1,2,29,30]. The formation of these heterocycles was the result of the presence of a chlorine atom at the C-4 position of the 1,2,3-dithiazole ring, which can be readily removed as a chloride anion. Presumably, one might expect that the exchange of 4-chlorine atom in 1,2,3-dithiazoles to poorly leaving groups, such as aryl or hetaryl, can significantly change reaction results. There is only one example of the benzoxazole ring formation (**5**) from 2-((4-(4-nitrophenyl)-5*H*-1,2,3-dithiazol-5-ylidene)amino)phenol **4** [28]. 4-Substituted 1,2,3-dithiazoles, except 4-chloro-1,2,3-dithiazoles, are much less available, and their chemistry still needs further developments. A couple of years ago, an easy one pot protocol for the preparation of the 4-substituted 1,2,3-dithiazolium chlorides from readily available acetoximes, disulfur dichloride and pyridine in MeCN has been developed [31]. The treatment of these salts was prepared in situ with aniline afforded 5-phenylimino-1,2,3-dithiazoles in low to moderate yields. Other 5-arylimino-1,2,3-dithiazoles including *o*-substituted derivatives, which can be used for the synthesis of new heterocyclic systems, were not obtained.

Herein, we report the synthesis of 2-((4-aryl(hetaryl)-5*H*-1,2,3-dithiazol-5-ylidene)amino)phenols, their thermal ring transformation into benzo[*d*]oxazol-2-yl(aryl(hetaryl)methanimines, followed by their unprecedented dimerization of into imidazole derivatives.

## 2. Results and Discussion

**Scheme 2:**
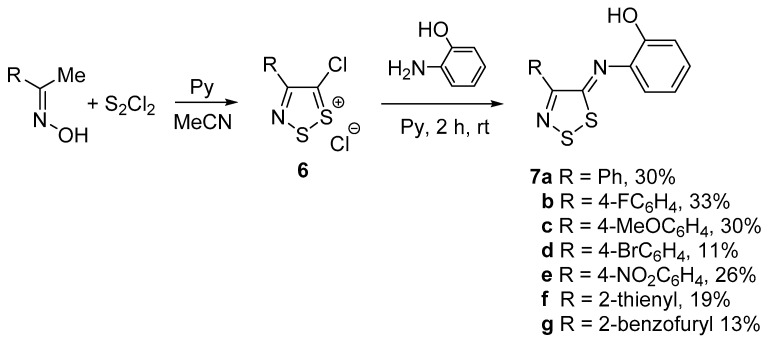
Synthesis of 2-((4-aryl(thien-2-yl)-5*H*-1,2,3-dithiazol-5-ylidene)amino)phenols **7**.

The treatment of 4-aryl(thienyl)-5-chloro-1,2,3-dithiazolium chlorides **6**, obtained in situ from acetoximes, disulfur dichloride, and pyridine in MeCN in a conditions described by us earlier [31], with *o*-aminophenol and pyridine at 0 °C and further stirring at room temperature for 2 h, gave imines **6** in moderate to low yields (Scheme 2). All our attempts to increase the yield of imines **7** by varying the base (DABCO, *N*-ethyldiisopropylamine or o-aminophenol), temperature of the reaction from −20 °C to room temperature, and time of dithiazolium salt **6** formation, resulted in a decrease in the yield of the target product to trace amounts. The low yields of imines **7** can be explained by low stability of the dithiazolium salts **6** at room temperature.

**Scheme 3:**
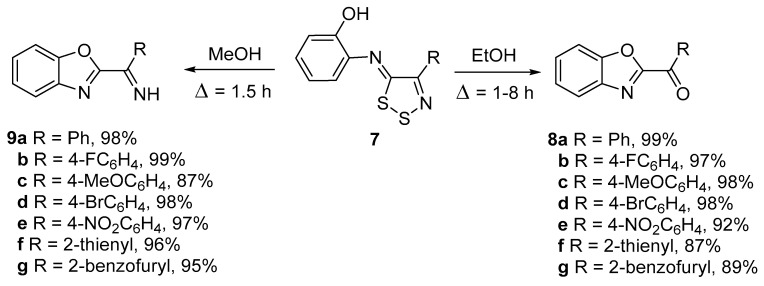
Thermolysis of 2-((4-aryl(thien-2-yl)-5*H*-1,2,3-dithiazol-5-ylidene)amino)phenols **7** in ethanol and methanol.

The thermal behavior of imines **7** was investigated in various solvents (chloroform, benzene, toluene, methanol, ethanol, acetonitrile). The imines **7** were found to be inert by prolonged refluxing (8 h) in chloroform (bp 61 °C) and benzene (bp 80 °C) and were isolated from the reaction mixtures in practically quantitative yields. The heating of the compounds in more polar solvent—ethanol (95% or anhydrous) afforded (benzo[*d*]oxazol-2-yl)arylmethanones **8** in high yields (Scheme 3).

**Figure 1:**
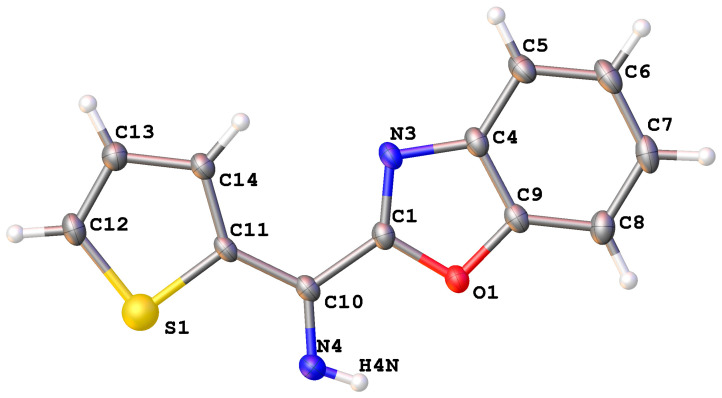
The general view of benzo[*d*]oxazol-2-yl(thien-2-yl)methanimine **9f** in representation of atoms by thermal ellipsoids (*p* = 50%).

When continuing the study of thermolysis of imines **7**, it was found that when heated in methanol (bp 65 °C), the formation of compounds other than methanones **8** is observed. The structure of the methanimines **9** has been confirmed by NMR and IR spectroscopy and mass-spectrometry and was unambiguously determined by an X-ray diffraction study of thienylmethanimine **9f** (Figure 1). Presumably, the formation of imines **9** and aroylbenzoxazoles **8** can be explained by the collapse of phenolic oxygen onto C-5 of the dithiazole ring with loss of HCl and sulfur followed by hydrolysis of methanimines **9 [28]**. The difference in the result of reactions in methanol and ethanol can be explained by the higher stability of imine **8** in methanol; for example, NMR spectra of imines **8** were successfully obtained in deuteromethanol, while our attempts to obtain similar spectra in deuteroethanol failed due to their decomposition (hydrolysis).

**Figure 2:**
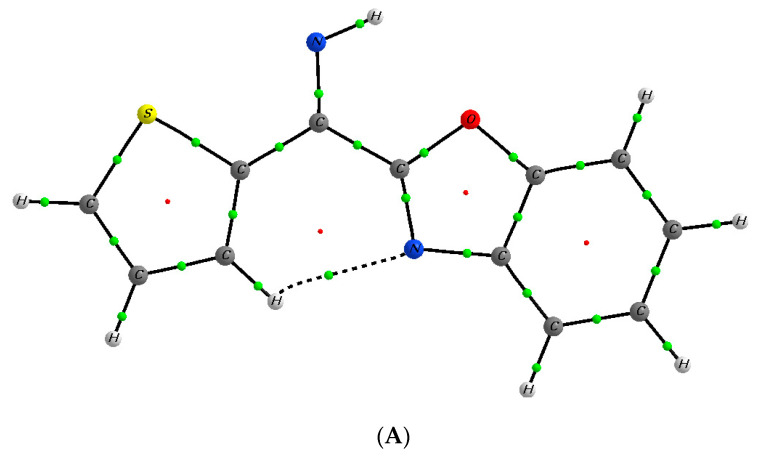

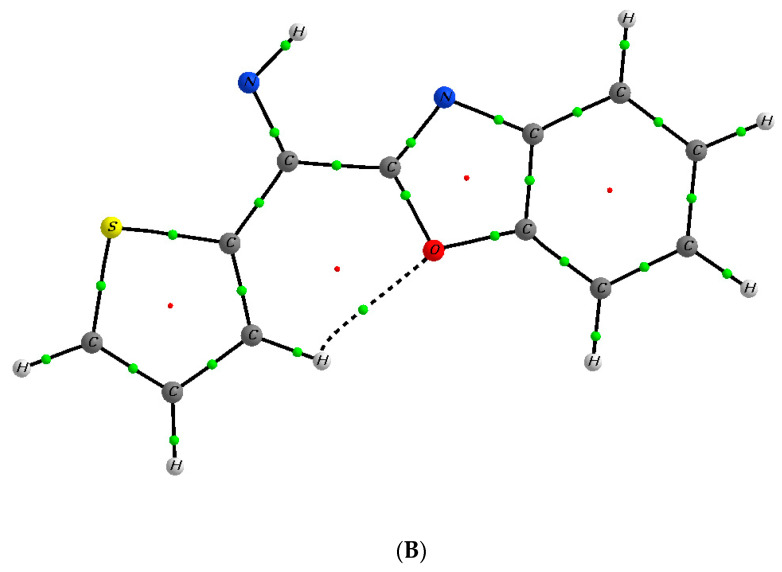
The molecular graphs of two isomers with trans- (**A**) and cis- (**B**) disposition of C=N bonds. The critical points (3, −1) and (3, +1) are shown by small green and red balls. The bond path for C-H···O and C-H···N interactions are shown by dashed lines.

In the crystal, **9f** exists in the form of an isomer with the imine group in transposition to the C=N bond of the benzo[*d*]oxazole ring. Such conformation can be stabilized by the intramolecular N4-H···O1 and/or C14-H···N3 interactions. In order to estimate the stability of two possible isomers, we performed PBE1PBE/def-2-TZVP calculations with the empirical dispersion corrections. The optimization at the above level was followed by the evaluation of the harmonic vibration frequencies. Full geometry optimization revealed that both isomers were characterized by almost equal energy with a small stabilization (0.87 kcal/mol) of the experimentally observed isomer (Figure 2).

The topological analysis of the electron density distribution function ρ(r) within Bader’s quantum theory of “Atoms in Molecule’’ (QTAIM) [32] revealed that in both isomers, the imine group did not participate in the formation of N-H···N or N-H···O interactions. In contrast, for C-H···O or C-H···N contacts, the critical points (3, −1) were located, and thus we can conclude that both of them are attractive interactions. The energy of the above intramolecular C-H···N and C-H···O interactions according to the correlation suggested by Espinosa et al. [33] was 3.1 and 2.9 kcal/mol.

Despite conjugation in the **9f**, the crystal molecule was not planar with the dihedral angle between the thiophen ring and rest molecule equal to 5.6°. Such conformation in **9f** is clearly the consequence of crystal packing. It was found out that the C=N-H group did not participate in any intermolecular hydrogen bond and that molecules were assembled by stacking interactions into infinite columns with the shortest C5···C10 contact equal to 3.405(2)Å.

**Scheme 4:**
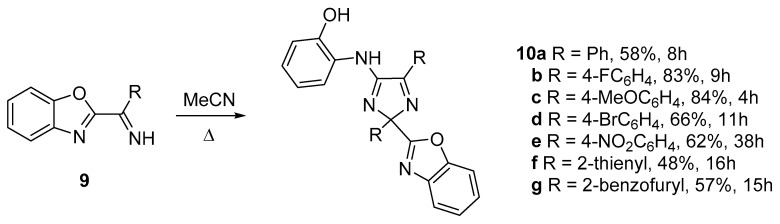
Synthesis of imidazoles **10** from methanimines **9**.

Further investigation of imines **7** thermolysis in toluene, acetonitrile, or THF showed the formation of new compounds **10** (TLC data) along with methanones **8** and methanimines **9**. This was confirmed by a prolonged (4–38 h) refluxing of methanimines **9** in MeCN, which led to the formation of products **10,** selectively, with good yields. Mass spectrometry, HRMS, and ^1^H and ^13^C NMR data showed that they are products of dimerization of methanimines **9** (Scheme 4).

**Figure 3:**
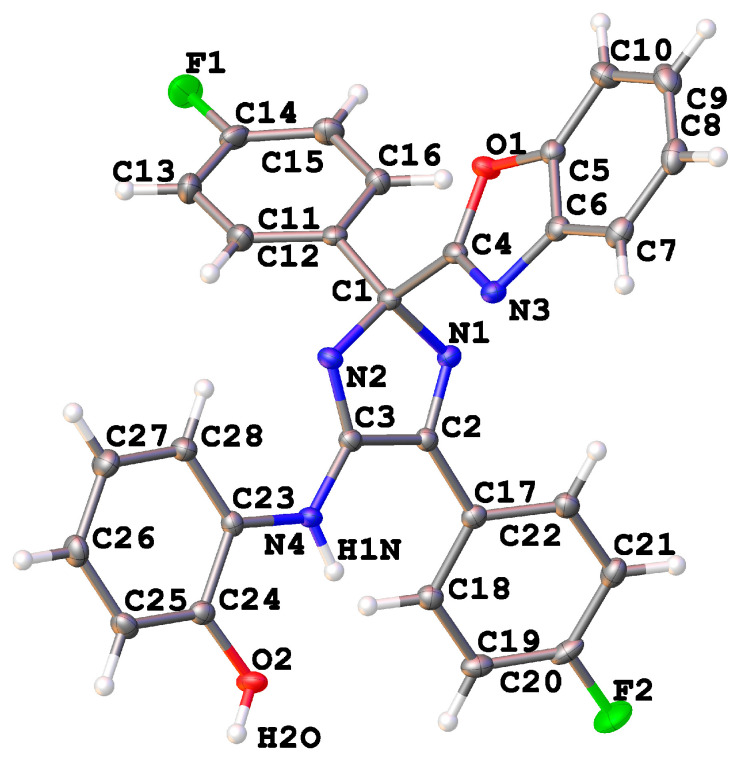
The general view of 2-((2-(benzo[*d*]oxazol-2-yl)-2,5-bis(4-fluorophenyl)-*2H*-imidazol-4-yl)amino)phenol **10b** in representation of atoms by thermal ellipsoids (*p* = 50%).

According to the literature search in SciFinder and Reaxys databases, no similar dimerization reaction of imino derivatives was published in the literature. The structure of imidazoles **10** was finally proved by the X-ray analysis for 4-fluorophenyl analogue **10b** (Figure 3).

**Figure 4:**
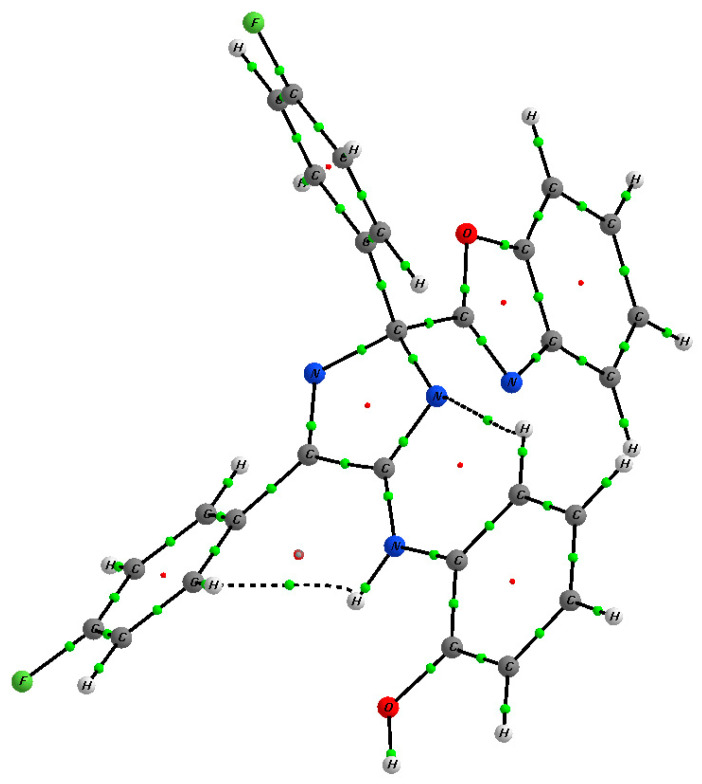
The molecular graph of **10b** according to PBE1PBE/def-2-TZVP calculation. The critical points (3, −1) and (3, +1) are shown by small green and red balls. The bond path for C-H···H-N and C-H···N interactions is shown by dashed lines.

The interesting feature of **10b** was the presence of the shortened N4-H···H-C18 intramolecular contact with H···H distance (with the account of C-H and N-H bond normalization) equal to 2.08Å. It is reasonable to propose that this shortened contact is clearly the consequence of the competition of the destabilization due to steric hindrance between atoms of amino-phenol and of fluorophenyl and stabilization due to conjugation of this substituents with the central 2*H*-imidazol ring. In order to analyze the nature of the observed NH···HC contact in the experimental conformation, we performed the optimization of hydrogen atom positions with all other parameters fixed. The consequent topological analysis of ρ(r) revealed that this shortened intramolecular contact corresponded to attractive interaction (Figure 4). Furthermore, the full optimization practically did not change the torsion angles, and the above H···H contact became as short as 2.068Å with CHH and NH···H angles equal to 116.2 and 96.1°. Thus, we can conclude that the observed conformation was not the consequence of the crystal packing effect but rather the inherent feature of this molecule.

**Figure 5:**
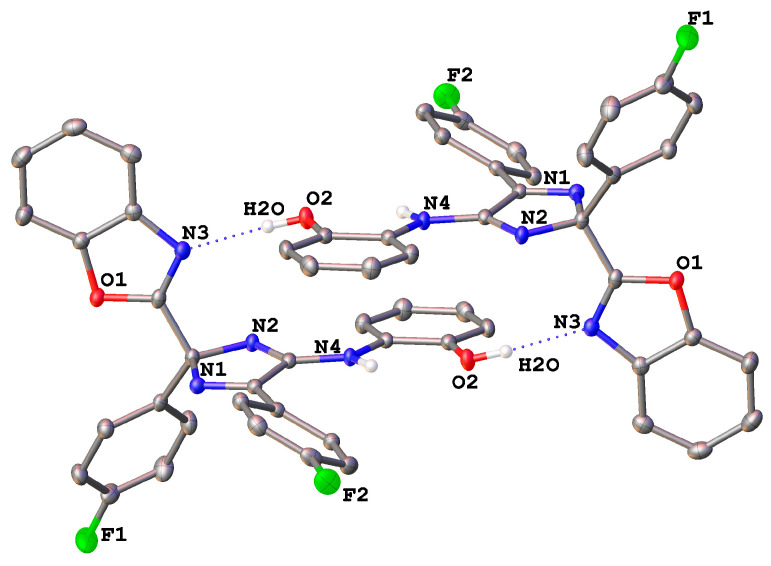
O-H···N and stacking bonded centrosymmetric dimer in the crystal of **10b**.

Crystal molecules were assembled into a centrosymmetric dimer due to the formation of the O2-H···N3 (O···N 2.840(2)Å) hydrogen bonds. The latter dimer was additionally stabilized by the stacking interactions with the interplane distance of ~3.4 Å (Figure 5).

### Mechanistic Rationale

**Scheme 5:**
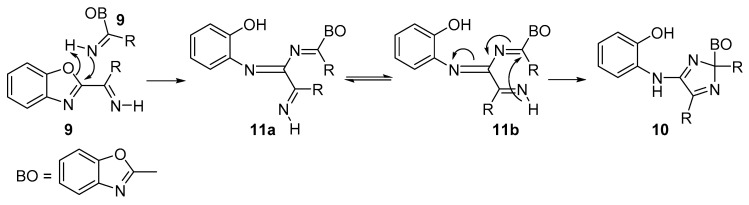
A suggested reaction path for the formation of imidazoles **9** from methanimines **8**.

The described procedure provides a new synthetic pathway to imidazole derivatives from compounds **9** containing methanimine and benzoxazole fragments. To the best of our knowledge, this reaction has not been described so far. We assume that the first step is the nucleophilic attack of the *N*-methanimine atom into the carbon atom of the oxazole ring with the opening of the benzoxazole ring to *o*-aminophenol moiety, which is well described for many benzoxazoles [34,35,36]. The result of this reaction is the formation of compounds containing three consecutive methanimino groups **11**. According to the search from Reaxys database, such structures were not known. The second intramolecular nucleophilic attack of the methanimino-nitrogen to the carbon atom of another methanimino group led to the 2-aminoimidazole ring closure (Scheme 5).

## 3. Experimental Section

### 3.1. General Methods and Materials

The reagents were purchased from commercial sources and used as received. Ethan-1-one oximes were prepared according to the published methods [37] and characterized by NMR spectra. All synthetic operations were performed under a dry argon atmosphere. Solvents were purified by distillation from the appropriate drying agents. Elemental analyses were performed on a 2400 Elemental Analyzer (Perkin Elmer Inc., Waltham, MA, USA). Melting points were determined on a Kofler hot-stage apparatus and were uncorrected. ^1^H and ^13^C NMR spectra were taken with a Bruker AM-300, AVANCE DRX 500, and AVANCE II 600 machines (Bruker Ltd., Moscow, Russia) with TMS as the standard. *J* values are given in Hz. MS spectra (EI, 70 eV) were obtained with a Finnigan MAT INCOS 50 instrument (Thermo Finnigan LLC, San Jose, CA, USA). High-resolution MS spectra were measured on a Bruker micrOTOF II instrument using electrospray ionization (ESI). The measurement was operated in a positive ion mode (interface capillary voltage −4500 V) or in a negative ion mode (3200 V); the mass range was from *m*/*z* 50 to *m*/*z* 3000 Da; external or internal calibration was performed with Electrospray Calibrant Solution (Fluka Chemicals Ltd., Gillingham, UK). A syringe injection was used for solutions in acetonitrile, methanol, or water (flow rate 3 μL·min^−1^). Nitrogen was applied as a dry gas; the interface temperature was set at 180 °C. IR spectra were measured with a Bruker “Alpha-T” instrument (Bruker, Billerica, MA, USA) in KBr pellets.

X-ray diffraction data for all studied compounds were collected using a SMART APEX II area-detector diffractometer (graphite monochromator, ω-scan technique) at the temperature of 120(2) K, using Mo_Kα_ radiation (0.71073 Å). The intensity data were integrated by the SAINT program and corrected for absorption and decay by the multiscan method (semi-empirical from equivalents) implemented in SADABS. All structures were solved by direct methods using SHELXS [38] and were refined against F^2^ using SHELXL-2017 [39]. All nonhydrogen atoms were refined with anisotropic displacement parameters. All C-H hydrogen atoms were placed in ideal calculated positions and refined as riding atoms with relative isotropic displacement parameters taken as *U*_iso_(H) = 1.2*U*_eq_(C). The hydrogen atoms of NH and OH groups were located from the Fourier density synthesis. Detailed crystallographic information is provided in Table 1 and as Supplementary Materials in CIF format that can be obtained free of charge via http://www.ccdc.cam.ac.uk/conts/retrieving.html or from the Cambridge Crystallographic Data Centre, 12, Union Road, Cambridge CB2 1EZ, UK; fax: 44-1223-336033 using the reference CCDC numbers (Table 1).

All quantum chemistry computations were performed in the Gaussian09 program [40] using the density functional theory (PBE0) [41] and the def-2-TZVP basis set. The choice of the PBE0 functional was based on the recent paper in which errors of various DFT functionals in the reproduction of an exact electron density and energy are discussed [42]. The geometry was optimized using the very tight optimization criteria and empirical dispersion corrections on the total energy [43] with the Becke-Johnson damping (D3) [44]. 

Topological analyses of the ρ(**r**) function were performed using the AIMAll program (AIMAll (Version 16.08.17), T. Keith, TK Gristmill Software, Overland Park KS, USA, 2016 (aim.tkgristmill.com)). All expected critical points were found, and the whole set of critical points in each system satisfies the Poincaré-Hopf rule. 

### 3.2. General Procedure for the Synthesis of 2-((4-aryl-5H-1,2,3-dithiazol-5-ylidene)amino)phenols (**7**)

Pyridine (0.24 mL, 3 mmol) was added dropwise at 0 to −5 °C to a stirred solution of ethanoneoxime **4** (1 mmol) and disulfur dichloride (0.16 mL, 2 mmol) in acetonitrile (10 mL) under inert atmosphere of argon. The mixture was stirred at 0 °C for 15–40 min. Then *o*-aminophenol (109 mg, 1 mmol) was added, the mixture was stirred at 0 °C for 30 min and followed by pyridine (0.16 mL, 2 mmol). The reaction mixture was stirred at room temperature for 2 h, filtered, and solvents were evaporated. The residue was separated by column chromatography (Silica gel Merck 60, light petroleum and then light petroleum–CH_2_Cl_2_ mixtures).

*2-((4-Phenyl-5H-1,2,3-dithiazol-5-ylidene)amino)phenol* (**7a**)

Yield 172 mg (30%). Yellow solid, m.p. 89–90 °C. Anal. calcd. for C_14_H_10_N_2_OS_2_: C, 58.72; H, 3.52; N, 9.78 found: C, 58.65; H, 3.56; N, 9.80. ^1^H NMR (300 MHz, CD_2_Cl_2_) *δ*: 8.27(s, 1H, Ar), 8.21 (d, 1H, *J* = 8.1, Ar), 7.74 (m, 3H, Ar), 7.57 (t, 1H, *J* = 7.3, Ar), 7.30 (d, 2H, *J* = 11.0, Ar), 7.15 (m, 1H, Ar), 7.03 (s, 1H, OH). ^13^C NMR (75 MHz, CD_2_Cl_2_) *δ*: 162.6, 161.9, 151.9, 134.7, 133.5, 130.7, 129.4, 129.1, 128.7, 120.1, 116.7, 114.8. IR, ν, cm^−1^: 3321, 3055, 1563, 1478, 1248, 1145, 1032, 722, 622. *m/z* (%): 286 (M^+^, 31), 222 (47), 119 (100), 91 (45). HRMS *m*/*z* (ESI) 287.0312 (calcd. for C_14_H_10_N_2_OS_2_ [M + H]^+^ 287.0312). 

*2-((4-(4-Fluorophenyl)-5H-1,2,3-dithiazol-5-ylidene)amino)phenol* (**7b**)

Yield 201 mg (33%). Yellow solid, m.p. 124–125 °C. Anal. calcd. for C_14_H_9_FN_2_OS_2_: C, 55.25; H, 2.98; N, 9.20 found: C, 55.20; H, 3.01; N, 9.24. ^1^H NMR (300 MHz, CD_2_Cl_2_) *δ*: 8.07 (m, 2H, Ar), 7.63 (d, 1H, *J* = 7.8, Ar), 7.25 (m, 3H, Ar), 7.06 (m, 2H, Ar), 6.53 (s, 1H, OH). ^13^C NMR (75 MHz, CD_2_Cl_2_) *δ*: 164.0 (*J* = 286), 161.4, 151.7, 134.9, 131.6, 131.5, 129.2, 120.2, 116.7, 116.0, 115.7, 115.0. IR, ν, cm^−1^: 3310, 1478, 1289, 1227, 1154, 1154, 862, 721. *m/z* (%): 304 (M^+^, 29), 240 (38), 183 (14), 119 (100), 91 (56). HRMS *m*/*z* (ESI) 305.0220 (calcd. for C_14_H_10_FN_2_OS_2_ [M + H]^+^ 305.0213).

*2-((4-(4-Methoxyphenyl)-5H-1,2,3-dithiazol-5-ylidene)amino)phenol* (**7c**)

Yield 190 mg (30%). Yellow solid, m.p. 137–138 °C. Anal. calcd. for C_15_H_12_N_2_O_2_S_2_: C, 56.94; H, 3.82; N, 8.85 found: C, 57.04; H, 3.78; N, 8.85.). ^1^H NMR (300 MHz, CD_2_Cl_2_) *δ*: 8.03 (d, 2H, *J* = 8.8, Ar), 7.64 (d, 1H, *J* = 7.7, Ar), 7.26 (t, 1H, *J* = 7.3, Ar), 7.06 (m, 4H, Ar), 6.63 (s, 1H, OH), 3.91 (s, 3H, CH_3_). ^13^C NMR (75 MHz, CD_2_Cl_2_) *δ*: 162.4, 161.9, 161.7, 151.7, 135.0, 130.9, 129.4, 128.9, 120.1, 116.7, 114.8, 114.1, 55.7. IR (KBr), ν, cm^−1^: 3376, 2836, 1609, 1479, 1313, 1250, 1172, 1032, 801, 727, 612. *m/z* (%): 316 (M^+^, 15), 252 (53), 183 (9), 133 (100), 119 (47), 91 (34). HRMS *m*/*z* (ESI)317.0417 (calcd. for C_15_H_13_N_2_O_2_S_2_ [M + H]^+^ 317.0413). 

*2-((4-(4-Bromophenyl)-5H-1,2,3-dithiazol-5-ylidene)amino)phenol* (**7d**)

Yield 80 mg (11%). Yellow solid, m.p. 152–153 °C. Anal. calcd. for C_14_H_9_BrN_2_OS_2_: C, 46.03; H, 2.48; N, 7.67 found: C, 45.95; H, 2.53; N, 7.71. ^1^H NMR (300 MHz, CD_2_Cl_2_) *δ*: 7.95 (d, 2H, *J* = 8.1, Ar), 7.68 (d, 2H, *J* = 8.1, Ar), 7.61 (d, 1H, *J* = 8.1, Ar), 7.26 (t, 1H, *J* = 7.7, Ar), 7.06 (m, 2H), 6.54 (s, 1H, OH). ^13^C NMR (75 MHz, CD_2_Cl_2_) *δ*: 161.9, 161.2, 151.6, 134.9, 132.3, 131.9, 130.9, 129.1, 125.1, 120.1, 116.7, 115.0. IR, ν, cm^−1^: 3411, 3057, 1583, 1477, 1253, 1229, 1153, 1068, 1010, 851, 805, 772, 740, 725, 678. *m/z* (%): 366 (M^+^ +2, 11), 364 (M^+^, 9) 300 (12), 183 (27), 150 (5), 119 (100), 91 (47). HRMS *m*/*z* (ESI)366.9384 (calcd. for C_14_H_10_BrN_2_OS_2_ [M + H]^+^ 366.9384). 

*2-((4-(4-Nitrophenyl)-5H-1,2,3-dithiazol-5-ylidene)amino)phenol* (**7e**)

Yield 172 mg (26%). Orange solid, m.p. 140–141 °C. Anal. calcd. for C_14_H_9_N_3_O_3_S_2_: C, 50.74; H, 2.74; N, 12.68 found: C, 50.68; H, 2.76; N, 12.72. ^1^H NMR (300 MHz, CD_2_Cl_2_) *δ*: 8.37 (d, 2H, *J* = 8.9, Ar), 8.26 (d, 2H, *J* = 8.9, Ar), 7.61 (d, 1H, *J* = 8.0, Ar), 7.28 (t, 1H, *J* = 7.8, Ar), 7.11–7.03 (m, 2H, Ar), 6.40 (s, 1H, Ar). ^13^C NMR (75 MHz, CD_2_Cl_2_) *δ*: 161.8, 160.3, 151.5, 149.0, 139.1, 134.8, 130.5, 129.4, 123.9, 120.3, 116.6, 115.1. IR, ν, cm^−1^: 3395, 1560, 1480, 1218, 1152, 1042, 843, 756, 715, 696. *m/z* (%): 331 (M^+^, 41), 231 (25), 183 (15), 148 (100), 123 (14), 79 (9). HRMS *m*/*z* (ESI)332.0158 (calcd. for C_14_H_10_N_3_O_3_S_2_ [M + H]^+^ 332.0162). 

*2-((4-(Thiophen-2-yl)-5H-1,2,3-dithiazol-5-ylidene)amino)phenol* (**7f**)

Yield 111 mg (19%). Orange solid, m.p. 77–78 °C. Anal. calcd. for C_12_H_8_N_2_OS_3_: C, 49.29; H, 2.76; N, 9.58 found: C, 49.21; H, 2.80; N, 9.56. ^1^H NMR (300 MHz, CD_2_Cl_2_) *δ*: 8.17 (d, 1H, *J* = 5.1, Ar), 7.60 (m, 2H, Ar), 7.25 (m, 2H, Ar), 7.09 (m, 2H, Ar), 6.87 (s, 1H, OH). ^13^C NMR (75 MHz, CD_2_Cl_2_) *δ*: 161.5, 156.3, 152.3, 135.3, 134.4, 131.6, 130.2, 129.4, 127.9, 120.5, 116.9, 115.6. IR, ν, cm^−1^: 3439, 3119, 1556, 1479, 1222, 1151, 1035, 834, 775, 711, 680. *m/z* (%): 292 (M^+^, 32), 228 (46), 195 (16), 150 (7), 119 (100), 109 (58), 91 (47). HRMS *m*/*z* (ESI)292.9869 (calcd. for C_12_H_9_N_2_OS_3_ [M + H]^+^ 292.9872).

*2-((4-(Benzofuran-2-yl)-5H-1,2,3-dithiazol-5-ylidene)amino)phenol* (**7g**)

Yield 63 mg (13%). Orange solid, m.p. 167–169 °C. Anal. calcd. for C_16_H_10_N_2_O_2_S_2_: C, 58.88; H, 3.09; N, 8.58 found: C, 59.13; H, 3.26; N, 8.49. ^1^H NMR (600 MHz, CD_2_Cl_2_) *δ*: 8.04 (s, 1H, Ar), 7.75 (d, 1H, *J* = 8.1, Ar), 7.63 (d, 1H, *J* = 8.1, Ar), 7.54 (d, 1H, *J* = 8.1, Ar), 7.48 (t, 1H, *J* = 7.7, Ar), 7.35 (t, 1H, *J* = 7.3, Ar), 7.29 (t, 1H, *J* = 7.7, Ar), 7.09 (m, 2H, Ar), 6.50 (s, 1H, OH). ^13^C NMR (90 MHz, CD_2_Cl_2_) *δ*: 163.3, 156.0, 152.2, 151.1, 149.6, 137.1, 129.4, 128.3, 127.6, 124.4, 123.5, 121.0, 117.1, 115.7, 112.2, 110.5. IR, ν, cm^−1^: 3487, 3456, 3139, 3059, 2958, 2929, 2858, 1728, 1610, 1560, 1485, 1333, 1288, 1257, 1220, 1175, 1162, 1072, 1034, 883, 741, 668. *m/z* (%): 326 (M^+^, 76), 262 (100), 245 (36), 143 (98), 119 (63) 91 (19), 64 (17). HRMS *m*/*z* (ESI)327.0254 [M + H]^+^ (calc. for C_16_H_10_N_2_O_2_S_2_, *m*/*z* 327.0256).

### 3.3. General Procedure for the Thermolysis of 2-((4-aryl(hetaryl)-5H-1,2,3-dithiazol-5-ylidene)amino)phenols **7** in Various Solvents

Dithiazole **7** (0.2 mmol) was refluxed in solvent (10 mL) up to its disappearance (TLC control) for the time given below. The reaction mixture was evaporated, and the residue was separated by column chromatography (Silica gel Merck 60, light petroleum and then light petroleum–CH_2_Cl_2_, then CH_2_Cl_2_).

*Benzo[d]oxazol-2-yl(phenyl)methanone* (**8a**) 

EtOH, 1.5 h, yield 44 mg (99%). Colorless solid, m.p. 72–73 °C. (m.p. 74–75 °C) [36]. The ^1^H and ^13^C NMR spectra were similar to those samples prepared by the literature method [45].

*Benzo[d]oxazol-2-yl(4-fluorophenyl)methanone* (**8b**)

EtOH, 1 h, yield 47 mg (97%). Colorless solid, m.p. 138–141 °C. (m.p. 108–110 °C) [36]. The ^1^H and ^13^C NMR spectra were similar to those described in the literature [45].

*Benzo[d]oxazol-2-yl(4-methoxyphenyl)methanone* (**8c**)

EtOH, 1 h, yield 50 mg (98%). Colorless solid, m.p. 123–124 °C. (m.p. 78–80 °C) [36]. The ^1^H and ^13^C NMR spectra were similar to those described in the literature [45].

*Benzo[d]oxazol-2-yl(4-bromophenyl)methanone* (**8d**)

EtOH, 1.5 h, yield 59 mg (98%). Light yellow solid, m.p. 113–115 °C. (m.p. 140–142 °C) [37]. The ^1^H and ^13^C NMR spectra were similar to those described in the literature [46].

*Benzo[d]oxazol-2-yl(4-nitrophenyl)methanone* (**8e**)

EtOH, 1.5 h, yield 49 mg (92%). Colorless solid, m.p. 160–161 °C. (m.p. 139–141 °C) [36]. The ^1^H and ^13^C NMR spectra were similar to those described in the literature [45].

*Benzo[d]oxazol-2-yl(thiophen-2-yl)methanone* (**8f**)

EtOH, 8 h, yield 40 mg (87%). Colorless solid, m.p. 119–120 °C. (m.p. 105–107 °C) [36]. The ^1^H and ^13^C NMR spectra were similar to those described in the literature [45].

*Benzo[d]oxazol-2-yl(benzofuran-2-yl)methanone* (**8g**)

EtOH, 5 h, yield 59 mg (89%). Colorless solid, m.p. 210–211 °C. Anal. calcd. for C_16_H_9_NO_3_: C, 73.00; H, 3.45; N, 5.32 found: C, 73.15; H, 3.56; N, 5.11. ^1^H NMR (600 MHz, CD_2_Cl_2_) *δ*: 8.75 (s, 1H, Ar), 8.02 (d, 1H, *J* = 7.9, Ar), 7.91 (d, 1H, *J* = 7.9, Ar), 7.80 (d, 1H, *J* = 7.9, Ar), 7.72 (d, 1H, *J* = 8.6, Ar), 7.65–7,58 (m, 2H, Ar), 7.56 (t, 1H, *J* = 7.6, Ar), 7.43 (t, 1H, *J* = 7.9, Ar). ^13^C NMR (150 MHz, CD_2_Cl_2_) *δ*: 169.0, 156.9, 156.8, 151.0, 150.7, 141.1, 129.9, 126.9, 127.6, 126.3, 124.6, 124.6, 122.6, 121.2, 112.8, 112.2. IR, ν, cm^−1^: 3440, 3139, 3105, 3065, 2960, 2926, 2855, 2361, 2342, 1657, 1613, 1542, 1528 1478, 1445, 1326, 1309, 1265, 1124, 1006, 991, 895, 737. *m/z* (EI) 263 (M^+^, 39), 145 (100), 118 (4), 89 (71), 28 (10). HRMS *m*/*z* (ESI)302.0215 [M + K]^+^ (calc. for C_16_H_9_KNO_3,_ *m*/*z* 302.0214).

*Benzo[d]oxazol-2-yl(phenyl)methanimine* (**9a**)

MeOH, 1.5 h, yield 43 mg (98%). Colorless amorphous solid, m.p. 40–42 °C. Anal. calcd. for C_14_H_10_N_2_O: C, 73.00; H, 3.45; N, 5.32 found: C, 73.15; H, 3.56; N, 5.11. ^1^H NMR (500 MHz, CD_3_OD) *δ*: 8.07(s, 1H, Ar), 7.85 (d, 1H, *J* = 7.3, Ar), 7.76–7.72 (m, 2H, Ar), 7.59(d, 1H, *J* = 7.3, Ar), 7.55–7.52 (m, 3H, Ar), 7.48 (t, 1H, *J* = 7.6, Ar), 7.38 (d, 1H, *J* = 6.1, Ar). ^13^C NMR (150 MHz, CD_3_OD) *δ*: 162.4, 150.3, 140.6, 133.7, 130.8, 129.2, 128.8, 127.7, 126.9, 125.6, 124.9, 110.7. IR, ν, cm^−1^ 3463, 3434, 2363, 2339, 1721, 1704, 1634, 1562, 1545, 1526, 1511, 1400, 1369, 1041, 1000, 966, 671, 571, 430. *m/z* (%): 222 (M^+^, 100), 119 (84), 104 (58), 91 (38), 77 (44). HRMS *m*/*z* (ESI)245.0688 [M + Na]^+^ (calcd. for C_14_H_10_NaN_2_O, *m*/*z* 245.0685).

*Benzo[d]oxazol-2-yl(4-fluorophenyl)methanimine* (**9b**)

MeOH, 1 h, yield 46 mg (99%). Colorless solid, m.p. 103–104 °C. Anal. calcd. for C_14_H_9_FN_2_O: C, 69.99; H, 3.78; N, 11.66 found: C, 70.23; H, 3.98; N, 11.82. ^1^H NMR (500 MHz, CD_3_OD) *δ*: 8.06 (s, 1H, Ar), 7.79 (d, 1H, *J* = 8.1, Ar), 7.69 (d, 1H, *J* = 8.1, Ar), 7.53–7.40 (m, 3H, Ar), 7.21 (t, 3H, *J* = 8.8, Ar). ^13^C NMR (125 MHz, CD_2_Cl_2_) *δ*: 169.9, 150.0, 139.9 (*J* = 173), 131.1, 131.0 (, 128.3, 127.0, 124.9, 124.1, 114.7, 114.5, 110.7. IR, ν, cm^−1^: 3435, 3270, 3045, 1602, 1507, 1451, 1414, 1381, 1334, 1230, 1188, 1158, 1104, 952, 843, 744, 633. *m/z* (%): 240 (M^+^,100), 122 (68), 119 (94), 95 (57), 75 (54). HRMS *m*/*z* (ESI)263.0587 [M + Na]^+^ (calcd. for C_14_H_9_NaFN_2_O, *m*/*z* 263.0591).

*Benzo[d]oxazol-2-yl(4-methoxyphenyl)methanimine* (**9c**)

MeOH, 1 h, yield 43 mg (87%). Colorless amorphous solid, m.p. 63–64 °C. Anal. calcd. for C_15_H_12_N_2_O_2_: C, 71.42; H, 4.79; N, 11.10 found: C, 71.55; H, 4.92; N, 4.52. ^1^H NMR (500 MHz, CD_3_OD) *δ*: 8.07 (s, 1H, Ar), 7.85(d, 1H, *J* = 7.9, Ar), 7.75 (d, 1H, *J* = 8.6, Ar), 7.55 (t, 1H, *J* = 7.3, Ar), 7.48(t, 1H, *J* = 7.3, Ar), 7.06 (d, 2H, *J* = 9.2, Ar) 3.89 (s, 3H, MeO). ^13^C NMR (125 MHz, CD_3_OD) *δ*: 162.4, 150.2, 140.5, 132.9, 130.4, 126.8, 124.9, 120.5, 119.1, 113.1, 112.8, 110.7, 54.2. IR, ν, cm^−1^ 3465, 3433, 3402, 3276, 3084, 3014, 2951, 2931, 2913, 2838, 2382, 2346, 2290, 1777, 1737, 1721, 1704, 1686, 1653, 1608, 1539, 1454, 1421, 1377, 1337, 1306, 1256, 1175, 1154, 1110, 1070, 1029, 1004, 951, 913, 893, 832, 808, 745, 712, 651, 625, 610, 563, 507, 427. *m/z* (%) 252 (M^+^,100), 237 (6), 221 (13), 134 (75), 119 (37), 91 (11), 77 (7). HRMS *m*/*z* (ESI)275.0791 [M + Na]^+^ (calcd. for C_15_H_12_NaN_2_O_2_, *m*/*z* 275.0791).

*Benzo[d]oxazol-2-yl(4-bromophenyl)methanimine* (**9d**)

MeOH.1.2 h, yield 60 mg (98%). Colorless solid, m.p. 82–83 °C. Anal. calcd. for C_14_H_9_BrN_2_O: C, 55.84; H, 3.01; N, 9.30 found: C, 55.99; H, 3.23; N, 9.11. ^1^H NMR (600 MHz, CD_3_OD) *δ*: 8.07 (d, 1H, *J* = 6.6, Ar), 7.88 (d, 1H, *J* = 8.1, Ar), 7.78 (d, 1H, *J* = 8.1, Ar), 7.73 (d, 2H, *J* = 8.8, Ar), 7.58 (t, 1H, *J* = 8.4, Ar), 7.51 (t, 1H, *J* = 7.3, Ar). ^13^C NMR (150 MHz, CD_2_Cl_2_) *δ*: 151.4, 141.7, 133.0, 132.0, 129.3, 128.2, 126.3, 126.1, 125.3, 121.8, 111.8, 111.3. IR, ν, cm^−1^: 3587, 3492, 3435, 2926, 2856, 1591, 1530, 1485, 1459, 1357, 1237, 1191, 1148, 1072, 1010, 945, 862, 826, 773, 738, 691. *m/z* (%): 299 (M^+^, 32%), 221 (7), 182 (21), 119 (100), 91 (43), 76 (25). HRMS *m*/*z* (ESI)322.9792 [M + Na]^+^ 324.9770 [M + Na]^+^ (calcd. for C_14_H_9_NaBrN_2_O, *m*/*z* 322.9792, 324.9770).

*Benzo[d]oxazol-2-yl(4-nitrophenyl)methanimine* (**9e**)

MeOH, 1.5 h, yield 60 mg (97%). Colorless solid, m.p. 130–131 °C. Anal. calcd. for C_14_H_9_N_3_O_3_: C, 62.92; H, 3.39; N, 15.72 found: C, 63.13; H, 3.52; N, 15.48. ^1^H NMR (600 MHz, CD_3_OD) 8.10–7.94 (m, 4H, Ar), 7.80 (d, 1H, *J* = 9.5, Ar), 7.72 (d, 1H, *J* = 5.9, Ar), 7.42–7.26 (m, 2H, Ar). ^13^C NMR (150 MHz, CD_2_Cl_2_) *δ*: 169.5, 152.0, 148.1, 147.9, 132.4, 132.2, 127.7, 124.4, 121.1, 119.5, 118.6, 110.6. IR, ν, cm^−1^: 3467, 3436, 3267, 3109, 1589, 1521, 1482, 1447, 1408, 1347, 1238, 1156, 1106, 952, 917, 852, 743, 682. *m/z* (%): 267 (M^+^, 64%), 221 (3), 149 (7), 119 (100), 103 (28), 76 (25), 46 (9). HRMS *m*/*z* (ESI)290.0536 [M + Na]^+^ (calcd. for C_14_H_9_NaN_3_O_3_, *m*/*z* 290.0536).

*Benzo[d]oxazol-2-yl(thiophen-2-yl)methanimine* (**9f**)

MeOH, 1.5 h, yield 43 mg (96%). Colorless solid, m.p. 69–70 °C. Anal. calcd. for C_12_H_8_N_2_OS: C, 63.14; H, 3.53; N, 12.27 found: C, 63.07; H, 3.55; N, 12.30. ^1^H NMR (300 MHz, CD_2_Cl_2_) *δ*: 11.04 (s, 1H), 8.42 (d, 1H, *J* = 1.4, Ar), 7.92 (d, 1H, *J* = 7.5, Ar), 7.72 (d, 1H, *J* = 7.9, Ar), 7.63 (d, 1H, *J* = 4.8, Ar), 7.61–7.44 (m, 2H, Ar), 7.25 (t, 1H, *J* = 4.3, Ar). ^13^C NMR (75 MHz, CD_2_Cl_2_) *δ*: 156.9, 150.8, 141.5, 137.8, 133.1, 131.1, 128.3, 127.6, 125.7, 121.8, 112.1, 111.5. IR, ν, cm^−1^: 3464, 3296, 3088, 2926, 1638, 1571, 1541, 1431, 1229, 1137, 1048, 942, 836, 744, 730, 611. *m/z* (%): 228 (M^+^, 77%), 144 (23), 120 (6), 109 (100), 84 (7), 64 (6), 76 (5). HRMS *m*/*z* (ESI) 229.0432 [M + H]^+^ (calcd. for C_12_H_9_N_2_OS, *m*/*z* 229.0430).

*Benzo[d]oxazol-2-yl(benzofuran-2-yl)methanimine* (**9g**)

MeOH, 1.5 h, yield 50 mg (95%). Colorless solid, m.p. 159–161 °C. Anal. calcd. for C_16_H_10_N_2_O_2_: C, 73.27; H, 3.84; N, 10.68 found: C, 73.45; H, 3.90; N, 10.42. NMR (600 MHz, CD_3_OD) *δ*: 8.11 (s, 1H, NH), 7.83 (d, 1H, *J* = 8.1, Ar), 7.70 (d, 1H, *J* = 7.3, Ar), 7.65 (d, 1H, *J* = 8.1, Ar), 7.53 (d, 1H, *J* = 8.1, Ar), 7.44 (t, 1H, *J* = 7.7, Ar), 7.39 (t, 2H, *J* = 7.7, Ar), 7.26 (t, 1H, *J* = 7.3, Ar). ^13^C NMR (90 MHz, CD_3_OD) *δ*: 154.7, 152.7, 149.8, 140.4, 128.8, 127.8, 126.7, 126.5, 124.6, 123.0, 120.6, 120.1, 111.7, 111.1, 110.9, 110.7. IR, ν, cm^−1^: 3448, 3287, 3138, 3092, 3065, 2957, 2924, 2853, 1654, 1615, 1590, 1557, 1525, 1474, 1450, 1229, 1170, 1123, 1005, 974, 894, 873, 734. *m/z* (%): 262 (M^+^, 100), 245 (24), 143 (81), 119 (51), 94 (13), 89 (50), 63 (34). HRMS *m*/*z* (ESI)301.0376 [M + K]^+^ (calcd. for C_16_H_10_N_2_O_2_K, *m*/*z* 301.0374).

*General procedure for the thermolysis of benzo[d]oxazol-2-yl(aryl(hetaryl))methanimines **9** in MeCN*

Methanimine **9** (0.2 mmol) was refluxed in MeCN (10 mL) up to its disappearance (TLC control) for the time given below. Reaction mixture was evaporated and the residue was separated by column chromatography (Silica gel Merck 60, light petroleum and then light petroleum–CH_2_Cl_2_, then CH_2_Cl_2_).

*2-((2-(Benzo[d]oxazol-2-yl)-2,5-diphenyl-2H-imidazol-4-yl)amino)phenol* (**10a**)

Yield 26 mg (58%). Colorless solid, m.p. 246–248 °C. Anal. calcd. for C_28_H_20_N_4_O_2_: C, 75.66; H, 4.54; N, 12.60 found: C, 75.70; H, 4.52; N, 12.56. ^1^H NMR (300 MHz, DMSO-*d*_6_,) *δ*: 10.26 (s, 1H, NH), 8.54 (d, 1H, *J* = 5.7, Ar), 8.02–7.90 (m, 5H, Ar), 7.71 (m, 5H, Ar), 7.47–7.41 (m, 5H, Ar), 6.94 (s, 3H, Ar). ^13^C NMR (125 MHz, DMSO-*d*_6_,) *δ*: 164.8, 163.8, 156.3, 150.3, 146.2, 140.3, 138.4, 131.7, 130.0, 129.6, 128.5, 128.3, 128.2, 128.1, 127.5, 125.8, 124.8, 123.6, 120.2, 119.5, 118.7, 114.5, 111.1, 100.8. IR, ν, cm^−1^: 3390, 3083, 1636, 1611, 1581, 1529, 1458, 1243, 746, 696, 568. *m/z* (%): 444 (M^+^, 12), 414 (26), 310 (9), 222 (100), 207 (48), 120 (16), 93 (41), 77 (12). HRMS *m*/*z* (ESI)445.1648 [M + H]^+^ (calc. for C_28_H_21_N_4_O_2_, *m*/*z* 445.1659).

*2-((2-(Benzo[d]oxazol-2-yl)-2,5-bis(4-fluorophenyl)-2H-imidazol-4-yl)amino)phenol* (**10b**)

Yield 40 mg (83%). Colorless solid, m.p. 217–219 °C. Anal. calcd. for C_28_H_18_F_2_N_4_O_2_: C, 69.99; H, 3.78; N, 11.66 found: C, 69.90; H, 3.82; N, 11.73. ^1^H NMR (300 MHz, DMSO-*d*_6_,) δ: 10.25 (s, 1H, NH), 8.46 (d, 1H, *J* = 7.4, Ar), 8.05 (s, 1H, OH), 8.03 (s, 2H, Ar), 7.96–7.91 (m, 2H, Ar), 7.76 (d, 1H, *J* = 8.7, Ar), 7.10 (d, 1H, *J* = 8.6, Ar), 7.53 (t, 2H, *J* = 8.7, Ar), 7.40–7.38 (m, 2H, Ar), 7.29 (t, 2H, *J* = 8.8, Ar), 6.95 (s, 3H, Ar). ^13^C NMR (125 MHz, DMSO-*d*_6_,) *δ*: 155.6 (*J* = 192), 155.5, 153.4 (*J* = 246), 147.8, 141.6, 137.8, 131.6, 125.8, 122.4, 121.7, 121.6, 118.7, 117.1, 116.1, 115.1, 111.5, 110.8, 110.4, 107.9, 106.5, 106.3, 106.0, 102.4, 91.4. IR, ν, cm^−1^: 3423, 3068, 2926, 1632, 1589, 1572, 1529, 1506, 1456, 1236, 1157, 1081, 832, 747, 524. *m/z* (%): 480 (M^+^, 75%), 359 (100), 346 (96), 239 (23), 225 (13), 197 (10), 122 (15), 91 (6). HRMS *m*/*z* (ESI)481.1477 [M + H]^+^ (calc. for C_28_H_19_F_2_N_4_O_2_, *m*/*z* 481.1471).

*2-((2-(Benzo[d]oxazol-2-yl)-2,5-bis(4-methoxyphenyl)-2H-imidazol-4-yl)amino)phenol* (**10c**)

Yield 42 mg (84%). Colorless crystals, m.p. 242–244 °C. Anal. calcd. for C_30_H_24_N_4_O_4_: C, 71.42; H, 4.79; N, 11.10 found: C, 71.50; H, 4.65; N, 11.05. ^1^H NMR (500 MHz, DMSO-*d*_6_,) δ: 10.28 (s, 1H, NH), 8.53 (d, 1H, *J* = 7.4, Ar), 7.99 (s, 1H, OH), 7.91 (d, 2H, *J* = 8.7, Ar), 7.79 (d, 2H, *J* = 8.8, Ar), 7.74 (d, 1H, *J* = 7.2, Ar), 7.69 (d, 1H, *J* = 7.2, Ar), 7.42–7.34 (m, 2H, Ar), 7.23 (d, 2H, *J* = 8.7, Ar), 7.01 (d, 2H, *J* = 8.8, Ar), 6.97–6.90 (m, 3H, Ar), 3.90 (s, 3H, CH_3_), 3.79 (s, 3H, CH_3_). ^13^C NMR (125 MHz, DMSO-*d*_6_,) *δ*: 155.5, 155.2, 153.1, 150.6, 147.5, 141.5, 137.4, 131.6, 121.9, 121.3, 120.7, 118.9, 116.9, 116.0, 114.7, 113.5, 111.4, 110.8, 110.0, 106.3, 105.8, 104.9, 102.3, 91.4, 46.8, 46.5. IR, ν, cm^−1^: 3391, 2927, 2840, 1608, 1582, 1509, 1458, 1250, 1172, 1026, 831, 747. *m/z* (%): 504 (M^+^, 9), 472 (25), 356 (82), 328 (37), 252 (100), 120 (5), 106(8), 93 (7), 78(14). HRMS *m*/*z* (ESI)505.1887 [M + H]^+^ (calc. for C_30_H_25_N_4_O_4_ *m*/*z* 505.1870).

*2-((2-(Benzo[d]oxazol-2-yl)-2,5-bis(4-bromophenyl)-2H-imidazol-4-yl)amino)phenol* (**10d**)

Yield 40 mg (66%). Colorless crystals, m.p. 251–253 °C. Anal. calcd. for C_28_H_18_Br_2_N_4_O_2_: C, 55.84; H, 3.01; N, 9.30 found: C, 55.84; H, 3.10; N, 9.25. ^1^H NMR (300 MHz, CD_2_Cl_2_) δ: 10.25 (s, 1H, NH), 7.85 (d, 2H, *J* = 8.2, Ar), 7.81–7.67 (m, 6H, Ar), 7.64–7.53 (m, 4H, Ar), 7.42–7.35 (m, 2H, Ar), 7.07 (t, 1H, *J* = 6.3, Ar), 6.96 (t, 2H, *J* = 7.2, Ar). ^13^C NMR (75 MHz, CD_2_Cl_2_) *δ*: 163.9, 163.4, 157.6, 151.4, 147.9, 147.8, 140.8, 137.3, 133.0, 132.7, 132.2, 131.8, 130.3, 130.2, 129.0, 126.3, 126.0, 125.0, 121.3, 121.0, 120.6, 111.2, 100.4. IR, ν, cm^−1^: 3407, 2925, 2854, 1635, 1591, 1579, 1457, 1243, 1072, 1010, 821, 748. *m/z* (%): 604 (M^+^ + 2, 13%), 602 (M^+^, 14), 522 (18), 483 (100), 446 (90), 442 (5), 300 (23), 284 (45), 167 (17), 156 (14), 107 (35), 79 (37). HRMS *m*/*z* (ESI)600.9875 [M + H]^+^ (calc. for C_28_H_19_Br_2_N_4_O_2_ *m*/*z* 600.9869).

*2-((2-(Benzo[d]oxazol-2-yl)-2,5-bis(4-nitrophenyl)-2H-imidazol-4-yl)amino)phenol* (**10e**)

Yield 40 mg (62%). Light yellow crystals, m.p. 213–215 °C. Anal. calcd. for C_28_H_18_N_6_O_6_: C, 62.92; H, 3.39; N, 15.72; O, 17.96 found: C, 62.85; H, 3.30; N, 15.81. ^1^H NMR (300 MHz, CD_2_Cl_2_,) 8.69 (d, 1H, *J* = 6.6), 8.35 (d, 2H, *J* = 8.1, Ar), 8.42–8.14 (m, 4H, Ar), 8.00 (t, 1H, *J* = 7.7, Ar), 7.89 (d, 2H, *J* = 8.1, Ar), 7.65 (d, 1H, *J* = 7.3, Ar), 7.47 (d, 1H, *J* = 7.3, Ar), 7.34–7.29 (m, 3H, Ar), 6.89 (t, 2H, *J* = 6.6, Ar), 6.78 (d, 1H, *J* = 4.4, Ar). ^13^C NMR (75 MHz, CD_2_Cl_2_,) *δ*: 163.7, 162.5, 156.9, 151.1, 149.8, 146.7, 144.1, 135.5, 129.7, 129.7, 126.1, 125.5, 125.0, 124.6, 123.5, 121.8, 121.2, 120.9, 120.4, 120.3, 116.9, 116.2, 111.1, 100.7. IR, ν, cm^−1^: 3420, 2956, 2927, 2855, 1776, 1736, 1720, 1703, 1685, 1639, 1583, 1521, 1458, 1406, 1347, 1311, 1282, 1245, 1218, 1201, 1173, 1107, 1076, 1039, 1014, 984, 932, 888, 849, 747, 692. *m/z* (%): 534 (M^+^, 38%), 488 (100), 435 (26), 411 (45), 365 (28), 268 (7), 148 (19), 123 (18), 79 (5). HRMS *m*/*z* (ESI)535.1366 [M + H]^+^ (calc. for C_28_H_19_N_6_O_6_, *m*/*z* 535.1361).

*2-((2-(Benzo[d]oxazol-2-yl)-2,5-bis(thiophen-2-yl)-2H-imidazol-4-yl)amino)phenol* (**10f**)

Yield 22 mg (48%). Dark brown amorphous crystals, m.p. 142–144 °C. Anal. calcd. for C_24_H_16_N_4_O_2_S_2_: C, 63.14; H, 3.53; N, 12.27 found: C, C, 63.11; H, 3.54; N, 12.32. ^1^H NMR (300 MHz, DMSO-*d*_6_,) 10.39 (s, 1H, NH), 8.42 (d, 1H, *J* = 7.4, Ar), 8.28 (s, 1H, OH), 8.08 (m, 2H, Ar), 8.04 (d, 1H, *J* = 3.5, Ar), 7.79–7.71 (m, 2H, Ar), 7.59 (d, 1H, *J* = 5.1, Ar), 7.45–7.40 (m, 4H, Ar), 7.13 (t, 1H, *J* = 8.7, Ar), 6.98–6.94 (m, 3H, Ar). ^13^C NMR (75 MHz, DMSO-*d*_6_,) *δ*: 158.4, 156.2, 150.2, 146.6, 140.2, 139.4, 132.8, 131.7, 130.5, 130.1, 129.1, 127.5, 127.3, 126.9, 126.7, 125.9, 124.8, 123.9, 121.9, 120.2, 119.4, 119.3, 114.6, 111.1. IR, ν, cm^−1^: 3392, 3116, 2927, 2855, 2362, 1629, 1577, 1531, 1457, 1384, 1239, 1067, 836, 748, 710. *m/z* (%): 456 (M^+^, 5%), 347 (21), 322 (50), 284 (100), 228 (18), 119 (19), 110 (15), 91 (9). HRMS *m*/*z* (ESI)457.0797 [M + H]^+^ (calc. for C_24_H_17_N_4_O_2_S_2_, *m*/*z* 457.0787).

*2-((2-(Benzo[d]oxazol-2-yl)-2,5-bis(benzofuran-2-yl)-2H-imidazol-4-yl)amino)phenol* (**10g**)

Yield 30 mg (57%). Yellow crystals, m.p. 231–233 °C. Anal. calcd. for C_32_H_20_N_4_O_4_: C, 73.45; H, 3.99; N, 10.32 found: C, 73.27; H, 3.84; N, 10.68. ^1^H NMR (300 MHz, CD_2_Cl_2_) *δ*: 8.94 (s, 1H, NH), 7.95 (d, 1H, *J* = 6.6, Ar), 7.89 (s, 1H, Ar), 7.83 (d, 1H, *J* = 7.3, Ar), 7.76 (d, 1H, *J* = 8.1, Ar), 7.64 (d, 1H, *J* = 7.3, Ar), 7.59–7.29 (m, 8H, Ar), 7.08 (s, 1H, Ar), 6.94 (d, 2H, *J* = 8.7, Ar), 6.85 (s, 1H, Ar). ^13^C NMR (150 MHz, CD_2_Cl_2_) *δ*: 161.9, 158.6, 155.8, 155.7, 152.0, 151.4, 147.7, 147.6, 140.8, 128.1, 127.9, 127.4, 127.3, 126.3, 125.9, 125.3, 125.2, 124.8, 123.5, 123.1, 121.9, 121.5, 120.9, 120.7, 120.0, 118.4, 116.8, 113.4, 112.2, 111.8, 111.4, 106.3. IR, ν, cm^−^^1^: 3386, 3115, 3064, 2923, 2853, 1632, 1592, 1557, 1506, 1455, 1348, 1281, 1248, 1166, 1147, 1106, 1068, 1002, 930, 874, 822, 746, 618, 430. *m/z* (%): 524 (M^+^, 16%), 262 (51), 145 (96), 119 (41), 94 (100), 63 (80). HRMS *m*/*z* (ESI)563.1116 [M + K]^+^ (calcd. for C_32_H_20_N_4_O_4_, *m*/*z* 563.1116).

## 4. Conclusions

In summary, a new unprecedented formation of four substituted imidazoles containing a benzoxazole ring from the thermolysis of readily available 2-((4-aryl(hetaryl)-5*H*-1,2,3-dithiazol-5-ylidene)amino)phenols was developed. The possibility of the imidazole ring formation from the compounds containing two methanimino groups was proved. Finally, 4-aryl(hetaryl)-substituted 5*H*-1,2,3-dithiazoles gave, upon thermolysis, different products from 4-chloro derivatives where the chlorine atom was readily expelled as a chloride anion, and the cyano group was generated. 2,2-Diaryl-2*H*-imidazol-4-amines are of interest as a BACE-1 inhibitors for the treatment of Alzheimer’s disease or dementia [47,48].

## Figures and Tables

**Table 1:** X-ray crystallographic data and refinement details for studied molecules.

	9f	10b
CCDC number	1993040	1993041
Empirical formula	C_28_H_18_F_2_N_4_O_2_	C_12_H_8_N_2_OS
Formula weight	480.46	228.26
T, K	120	120
Crystal system	Monoclinic	Monoclinic
Space group	P2_1_/*n*	P2_1_/*n*
Z (Z’)	4 (1)	4(1)
a, Å	12.3650(12)	6.1355(5)
b, Å	12.9901(13)	7.5454(6)
c, Å	14.3314(14)	22.0813(19)
α, °	90	90
β, °	107.871(2)	94.779(2)
γ, °	90	90
V, Å^3^	2190.9(4)	1018.70(15)
D_calc_,gcm^−3^	1.457	1.488
μ, cm^−1^	1.06	2.93
F(000)	992	472
2θ_max_, °	58	58
Reflections collected	25153	11900
Reflections unique (R_int_)	5821 (0.0410)	2710 (0.0357)
Reflections with I > 2σ(I)	4558	2443
Variables/restraints	333	149
R1	0.0425	0.0357
wR2	0.1092	0.0962
GOF	1.024	1.036
Largest difference in peak/hole (e/Å^3^)	0.328/−0.242	0.415/−0.342

## Supplementary Materials

The Supplementary Materials are available online. Characterization data including ^1^H and ^13^C NMR spectra for novel compounds and single crystal X-ray crystallography data (CCDC 1850211 and 1850212 for compounds **9f** and **10b**, respectively).
