# Supplementary material for: Synthesis of 2-((2-(Benzo[d]oxazol-2-yl)-2H-imidazol-4-yl)amino)-phenols from 2-((5H-1,2,3-Dithiazol-5-ylidene)amino)phenols through Unprecedented Formation of Imidazole Ring from Two Methanimino Groups
Source: Molecules. 2020 Aug 19;25(17):3768. doi: 10.3390/molecules25173768 (PMC7504547; doi:10.3390/molecules25173768)

Supplementary Materilas

Synthesis of 2-((2-(benzo[d]oxazol-2-yl)-2H-imidazol-4-yl)amino)-phenols from 2-((5H-1,2,3-dithiazol-5-ylidene)amino)phenols through unprecedented formation of imidazole ring from two methanimino groups

Ilia V. Baranovsky [1], Lidia S. Konstantinova [1,2], Mikhail A. Tolmachev [1], Vadim V. Popov [2], Konstantin A. Lyssenko [3,4], and Oleg A. Rakitin [1,2,*]

[1] N. D. Zelinsky Institute of Organic Chemistry, Russian Academy of Sciences, 119991 Moscow, Russia; orakitin@ioc.ac.ru (O.A.R.); konstantinova_ls@mail.ru (L.S.K.); ilay679@rambler.ru (I.V.B.); mtolmachev4@gmail.com (M.A.T.)

[2] Nanotechnology Education and Research Center, South Ural State University, 454080 Chelyabinsk, Russia; rakitinoa@susu.ru (O.A.R.); popov.ioc@gmail.com (V.V.P.)

[3] M.V. Lomonosov Moscow State University, Chemistry Department, Leninskiye Gory, 1, Moscow 119991, Russia; kostya@ineos.ac.ru (K.A.L.)

[4] G.V. Plekhanov Russian University of Economics, 36 Stremyanny Per., Moscow 117997, Russia; kostya@ineos.ac.ru (K.A.L.)

Fig. 1. ^{1}H NMR spectrum of 2-((4-phenyl-5H-1,2,3-dithiazol-5-ylidene)amino)phenol (**7a**) (300 MHz, CD_2Cl_2)

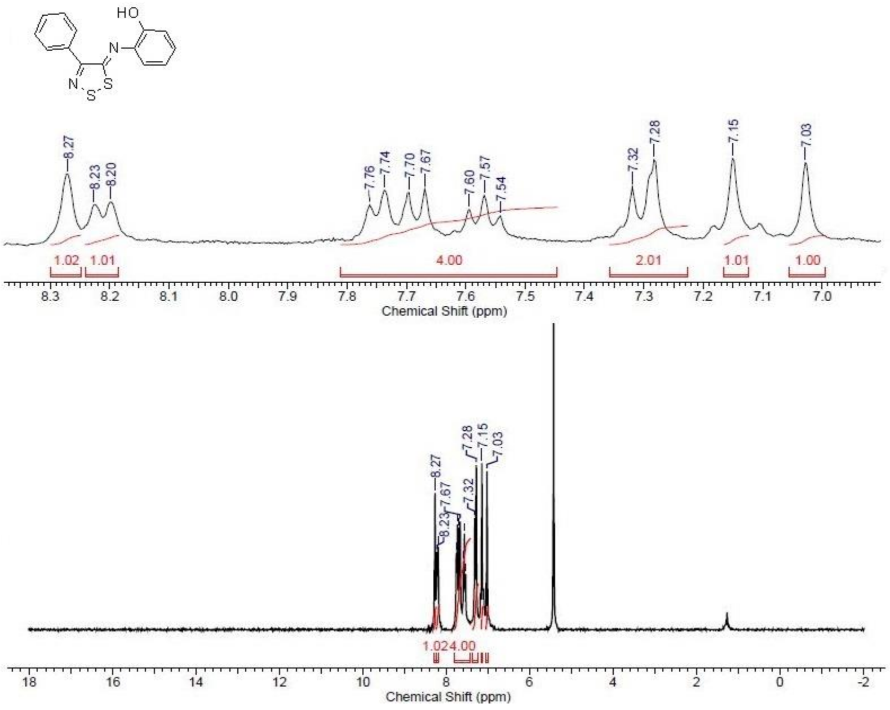

Fig. 2. ^{13}C NMR spectrum of 2-((4-phenyl-5H-1,2,3-dithiazol-5-ylidene)amino)phenol (**7a**) (300 MHz, CD_2Cl_2)

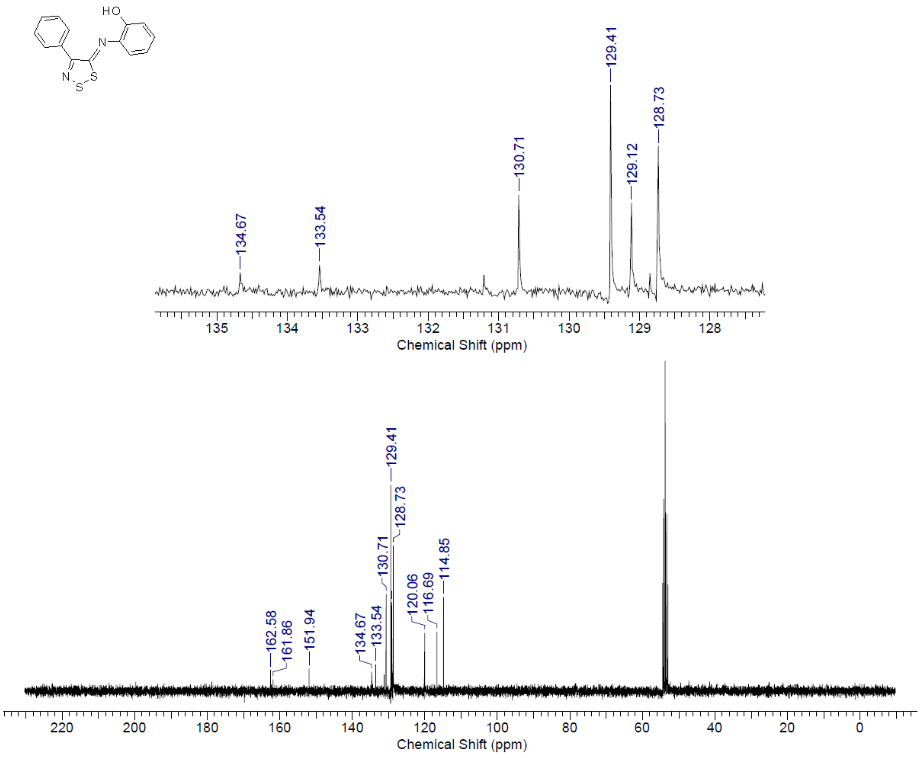

Fig. 3. ^{1}H NMR spectrum of 2-((4-(4-fluorophenyl)-5H-1,2,3-dithiazol-5-ylidene)amino)phenol (**7b**) (75 MHz, CD_2Cl_2)

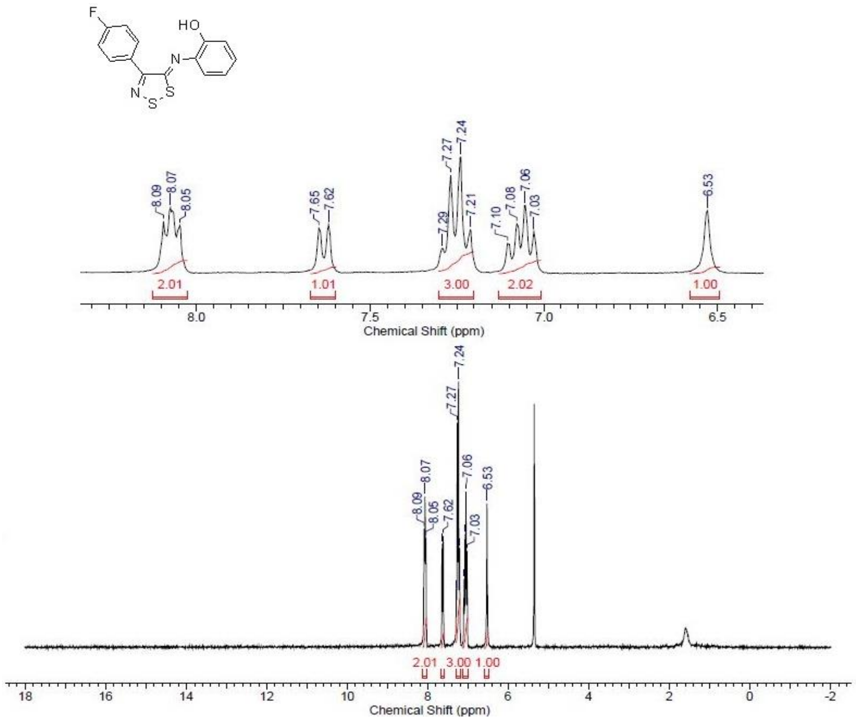

Fig. 4.^{13}C NMR spectrum of 2-((4-(4-fluorophenyl)-5H-1,2,3-dithiazol-5-ylidene)amino)phenol (**7b**) (75 MHz, CD_2Cl_2)

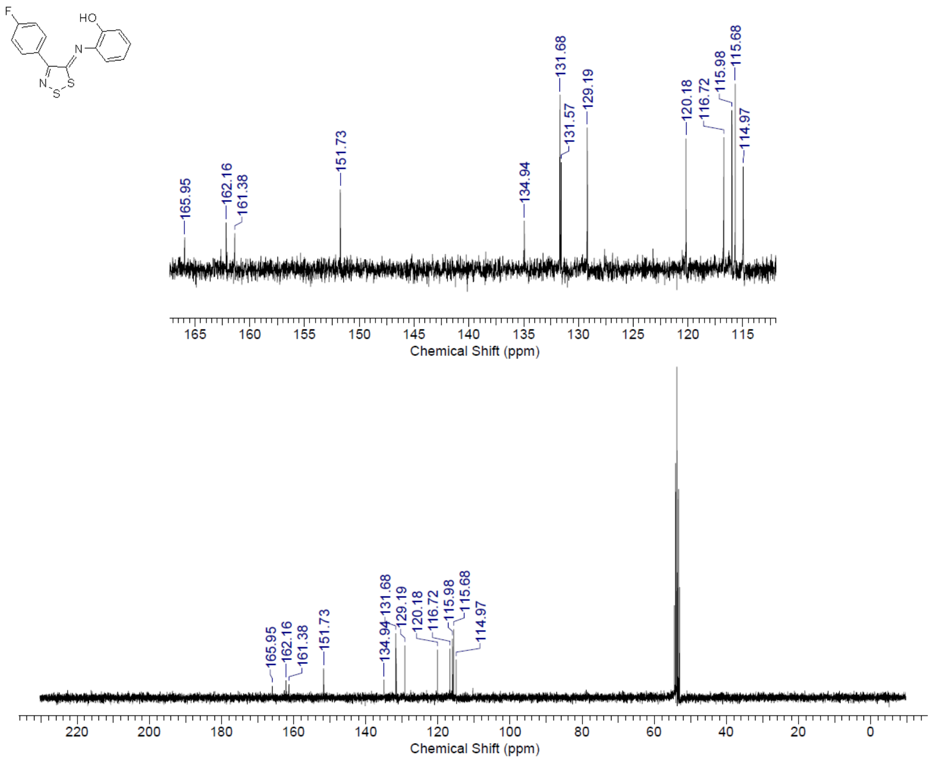

Fig. 5. ^{1}H NMR spectrum of 2-((4-(4-methoxyphenyl)-5H-1,2,3-dithiazol-5-ylidene)amino)phenol (**7c**) (300 MHz, CD_2Cl_2)

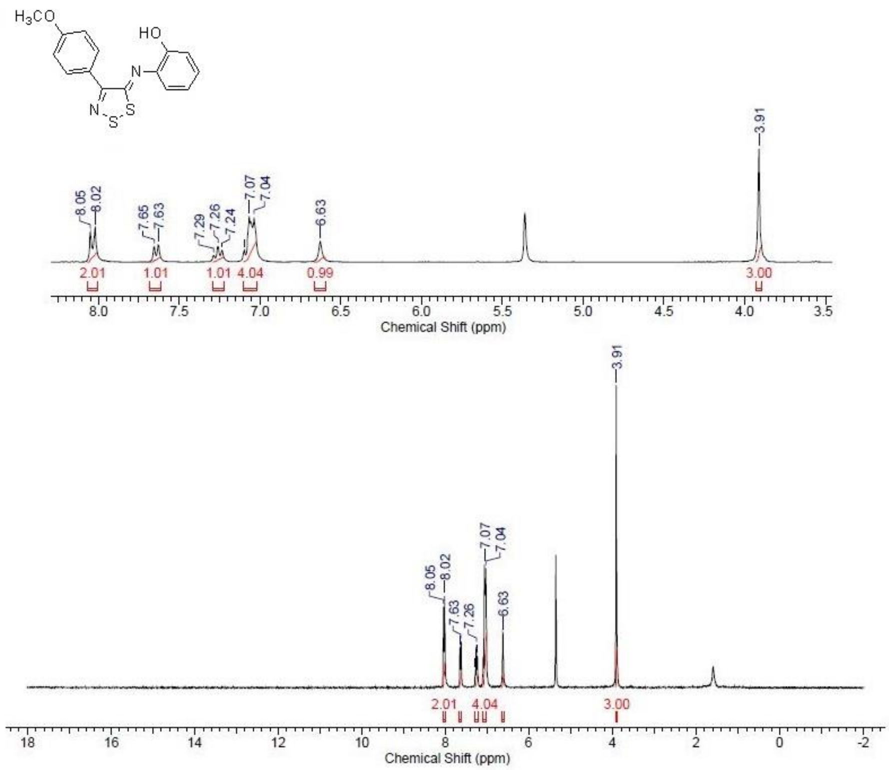

Fig. 6. ^{13}C NMR spectrum of 2-((4-(4-methoxyphenyl)-5H-1,2,3-dithiazol-5-ylidene)amino)phenol (**7c**) (75 MHz, CD_2Cl_2)

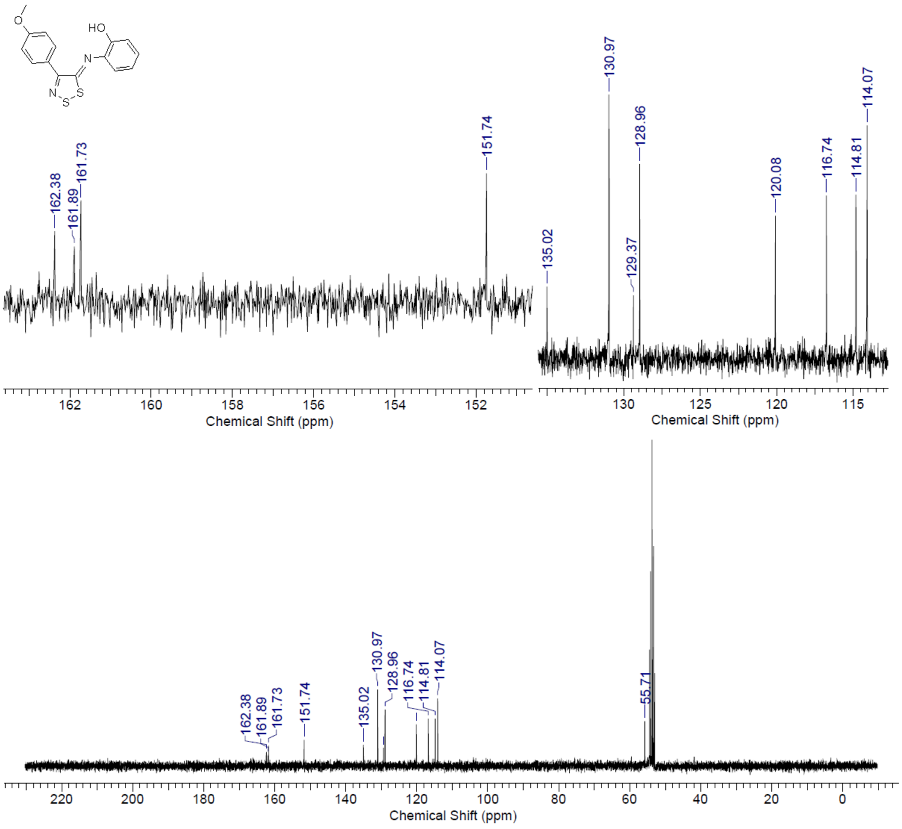

Fig. 7. ^{1}H NMR spectrum of 2-((4-(4-bromophenyl)-5H-1,2,3-dithiazol-5-ylidene)amino)phenol (**7d**) (300 MHz, CD_2Cl_2)

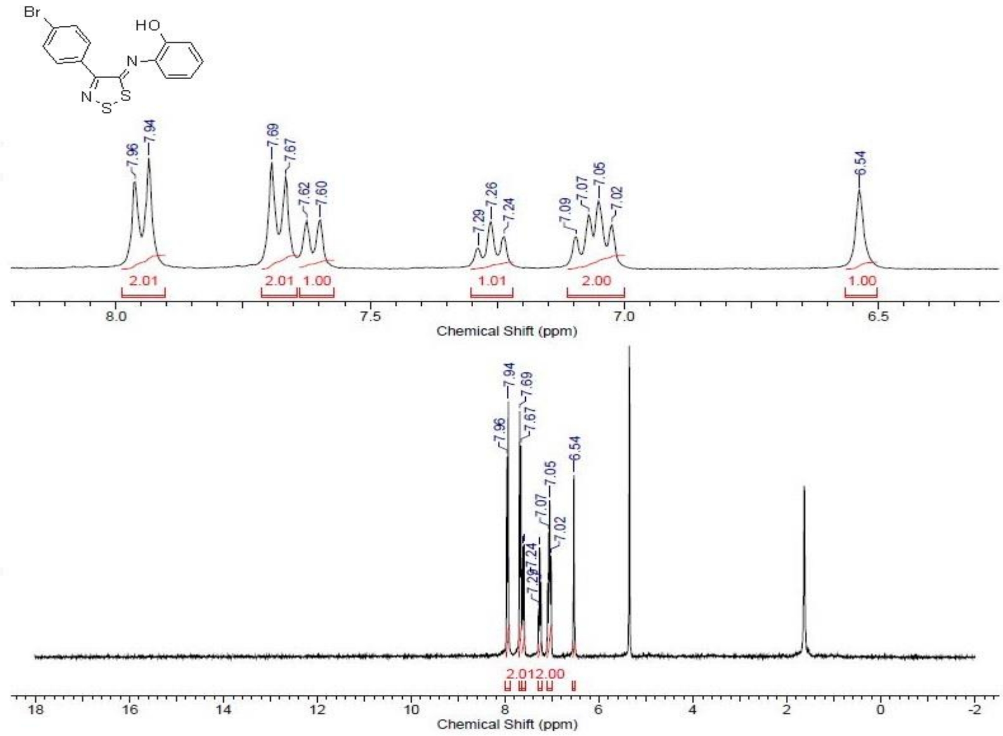

Fig. 8. ^{13}C NMR spectrum of 2-((4-(4-bromophenyl)-5H-1,2,3-dithiazol-5-ylidene)amino)phenol (**7d**) (150 MHz, CD_2Cl_2)

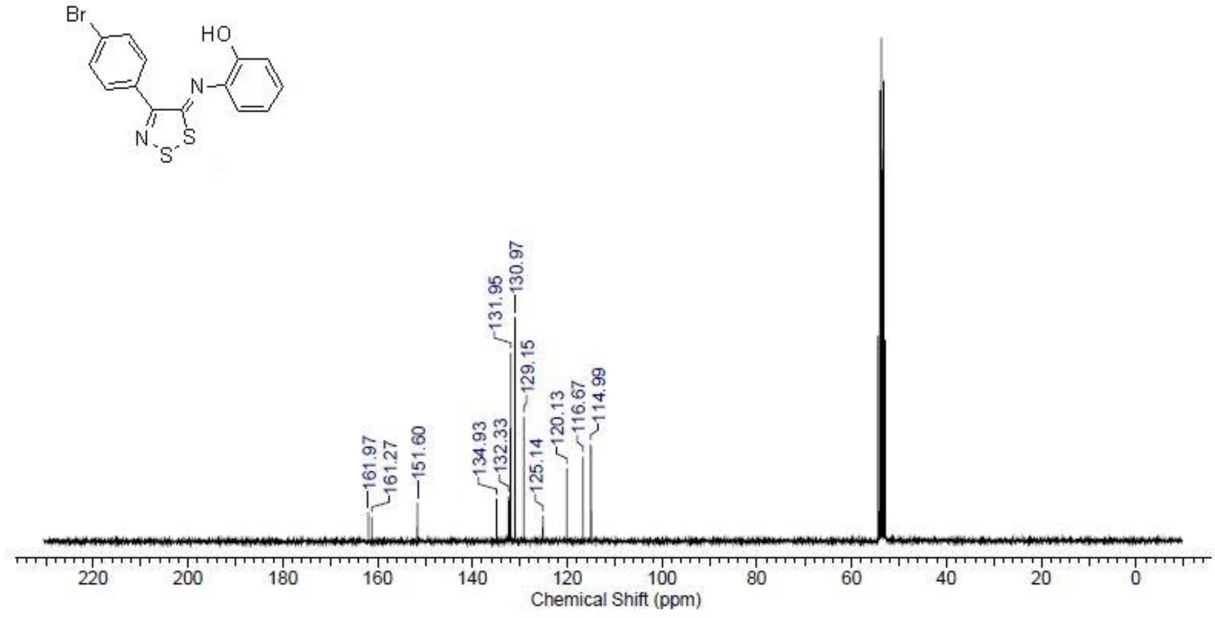

Fig. 9. ^{1}H NMR spectrum of 2-((4-(4-nitrophenyl)-5H-1,2,3-dithiazol-5-ylidene)amino)phenol (**7e**) (300 MHz, CD_2Cl_2)

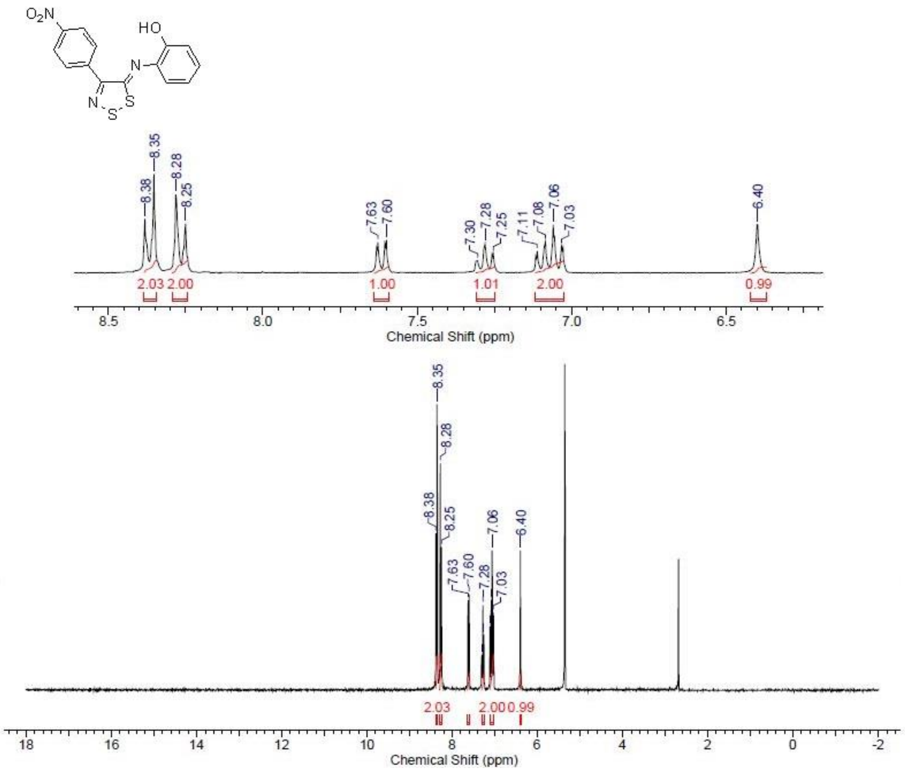

Fig. 10. ^{13}C NMR spectrum of 2-((4-(4-nitrophenyl)-5H-1,2,3-dithiazol-5-ylidene)amino)phenol (**7e**) (150 MHz, CD_2Cl_2)

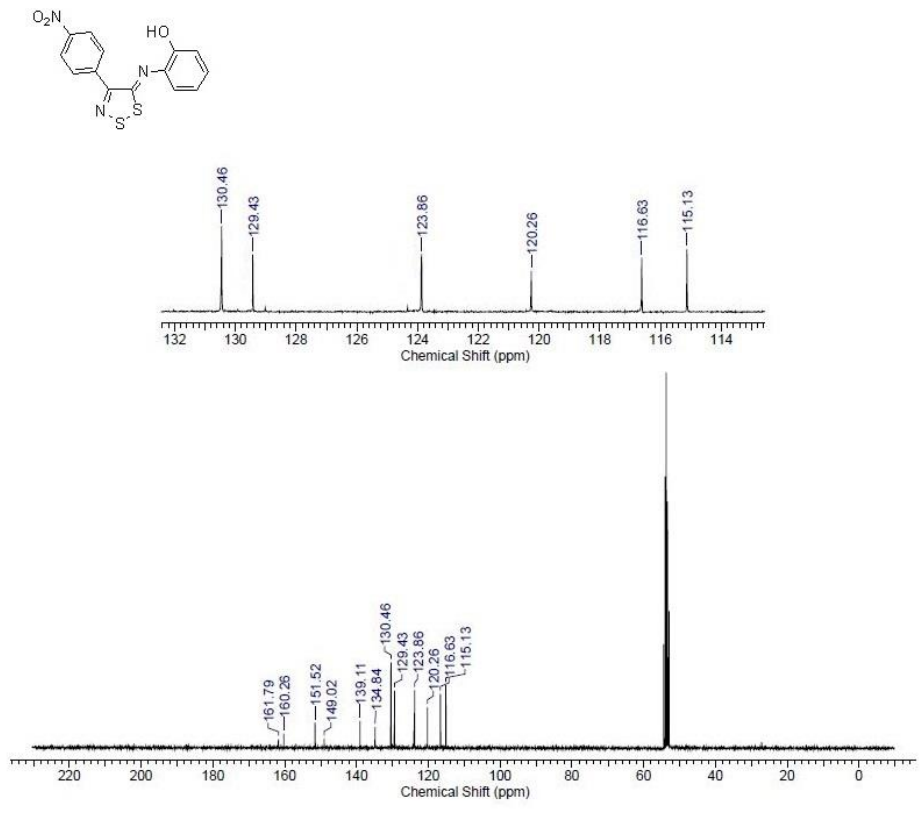

Fig. 11. ^{1}H NMR spectrum of 2-((4-(thiophen-2-yl)-5H-1,2,3-dithiazol-5-ylidene)amino)phenol (**7f**) (300 MHz, CD_2Cl_2)

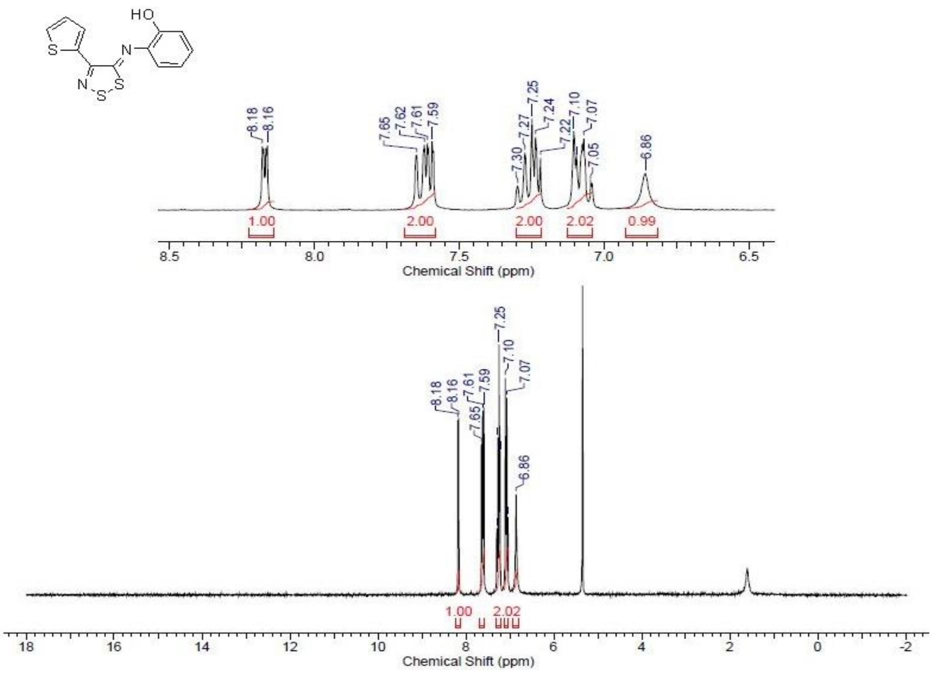

Fig. 12. ^{13}C NMR spectrum of 2-((4-(thiophen-2-yl)-5H-1,2,3-dithiazol-5-ylidene)amino)phenol (**7f**) (75 MHz, CD_2Cl_2)

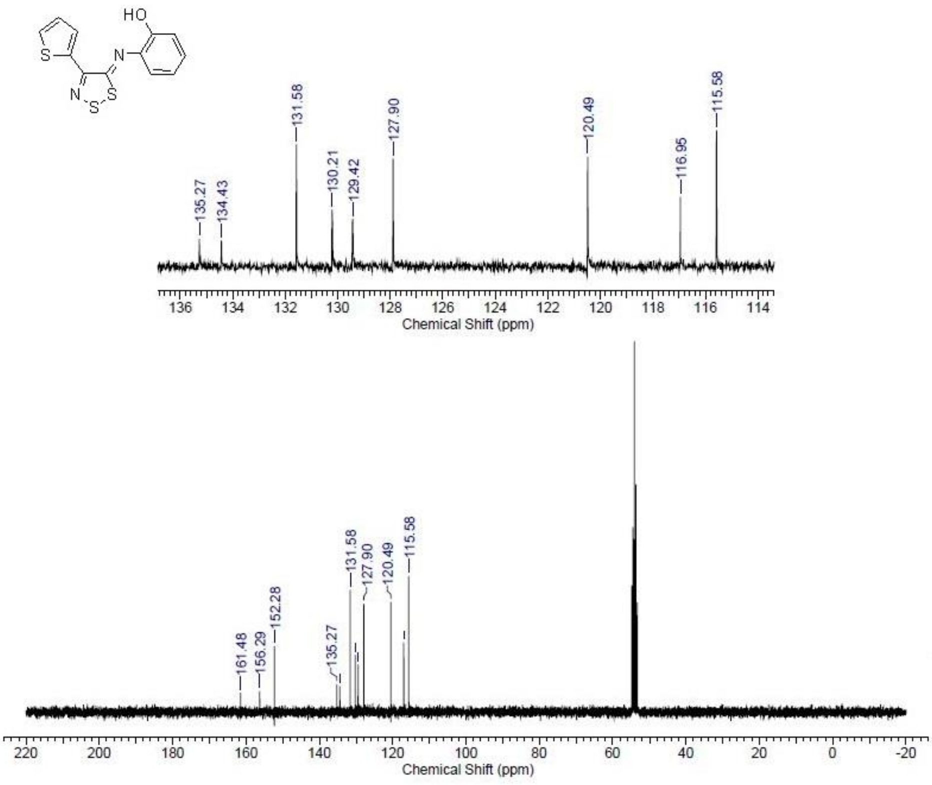

Fig. 13. ^{1}H NMR spectrum of 2-((4-(benzofuran-2-yl)-5H-1,2,3-dithiazol-5-ylidene)amino)phenol (**7g**) (300 MHz, CD_2Cl_2)

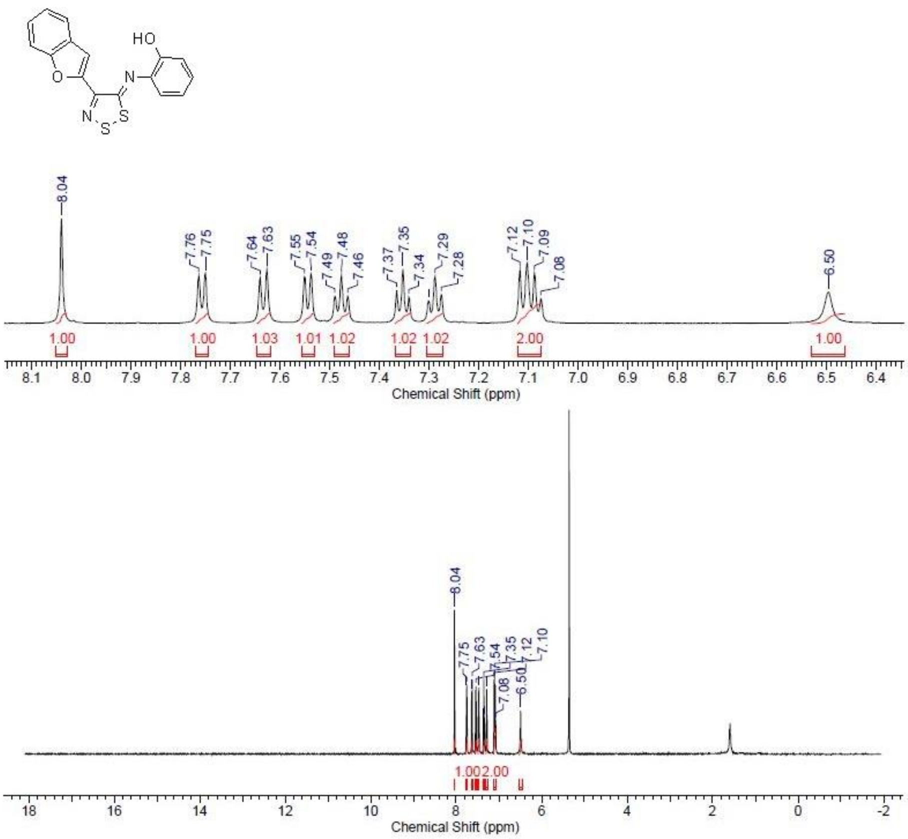

Fig. 14. ^{13}C NMR spectrum of 2-((4-(benzofuran-2-yl)-5H-1,2,3-dithiazol-5-ylidene)amino)phenol (**7g**) (125 MHz, CD_2Cl_2)

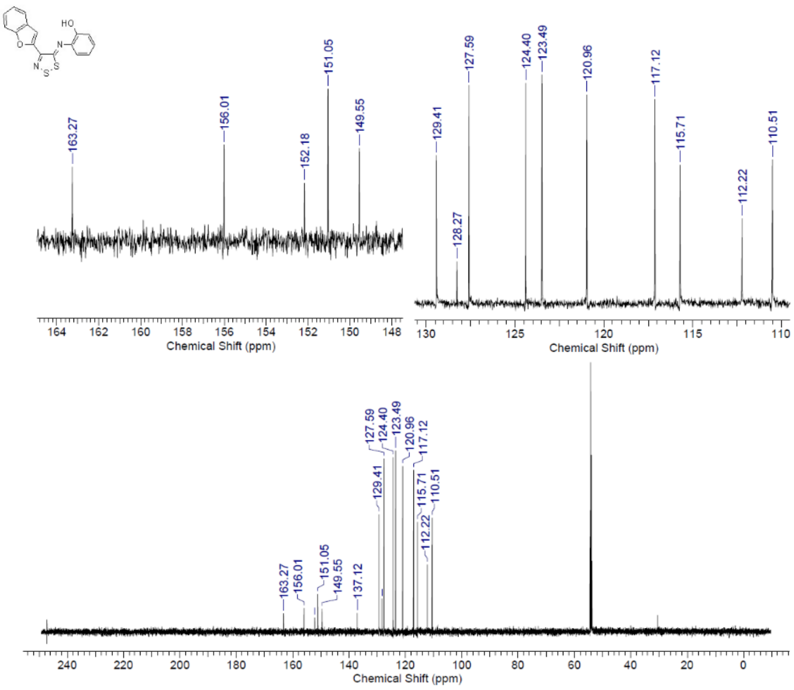

Fig. 15. ^{1}H NMR spectrum of benzo[d]oxazol-2-yl(phenyl)methanone (**8a**) (300 MHz, CD_2Cl_2)

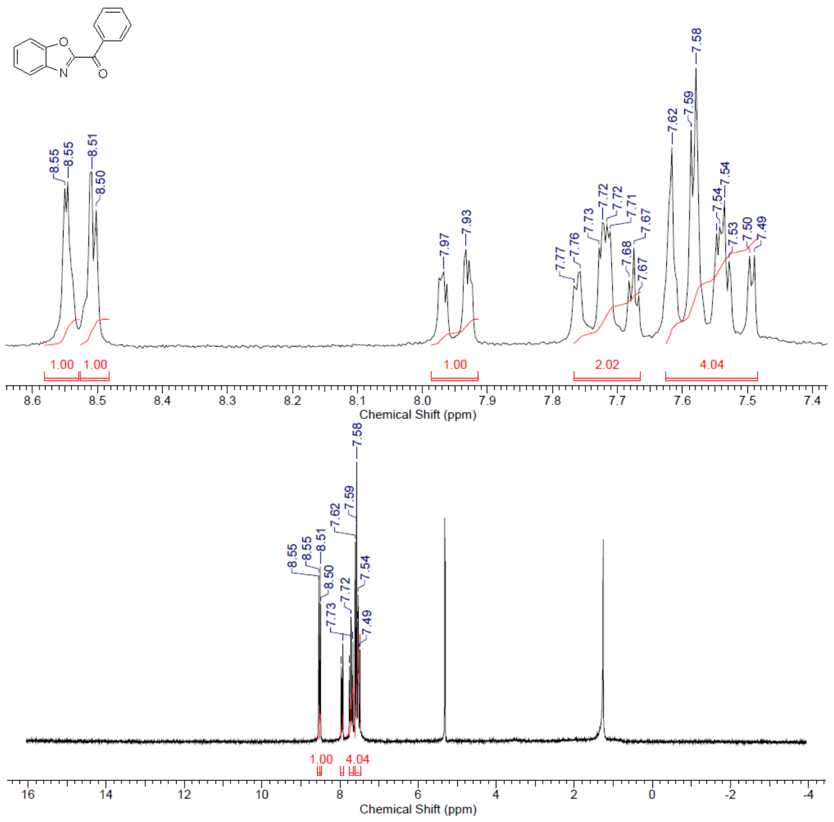

Fig. 16. ^{13}C NMR spectrum of benzo[d]oxazol-2-yl(phenyl)methanone (**8a**) (150 MHz, CD_2Cl_2)

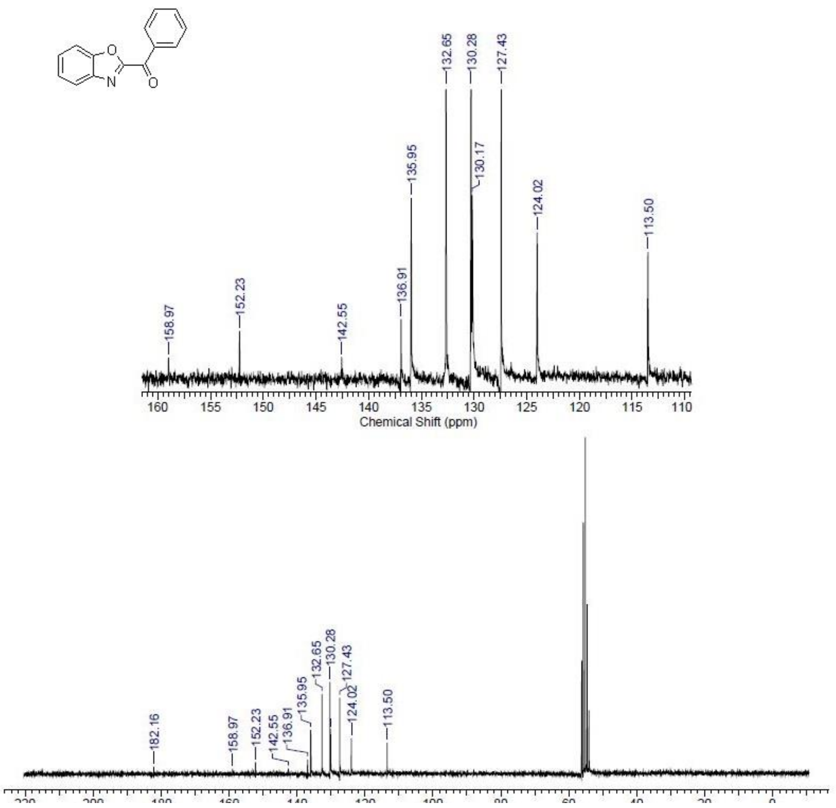

Fig. 17. ^{1}H NMR spectrum of benzo[d]oxazol-2-yl(4-fluorophenyl)methanone (**8b**) (300 MHz, CD_2Cl_2)

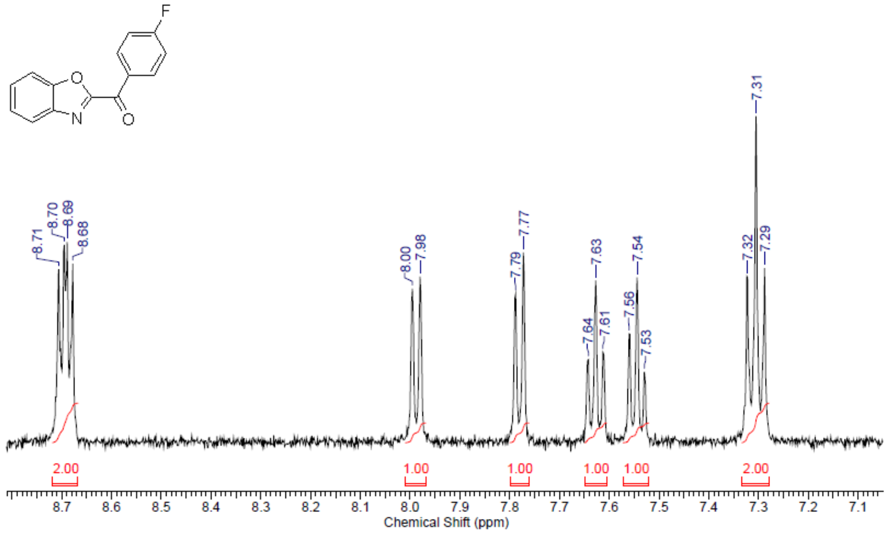

Fig. 18. ^{13}C NMR spectrum of benzo[d]oxazol-2-yl(4-fluorophenyl)methanone (**8b**) (150 MHz, CD_2Cl_2)

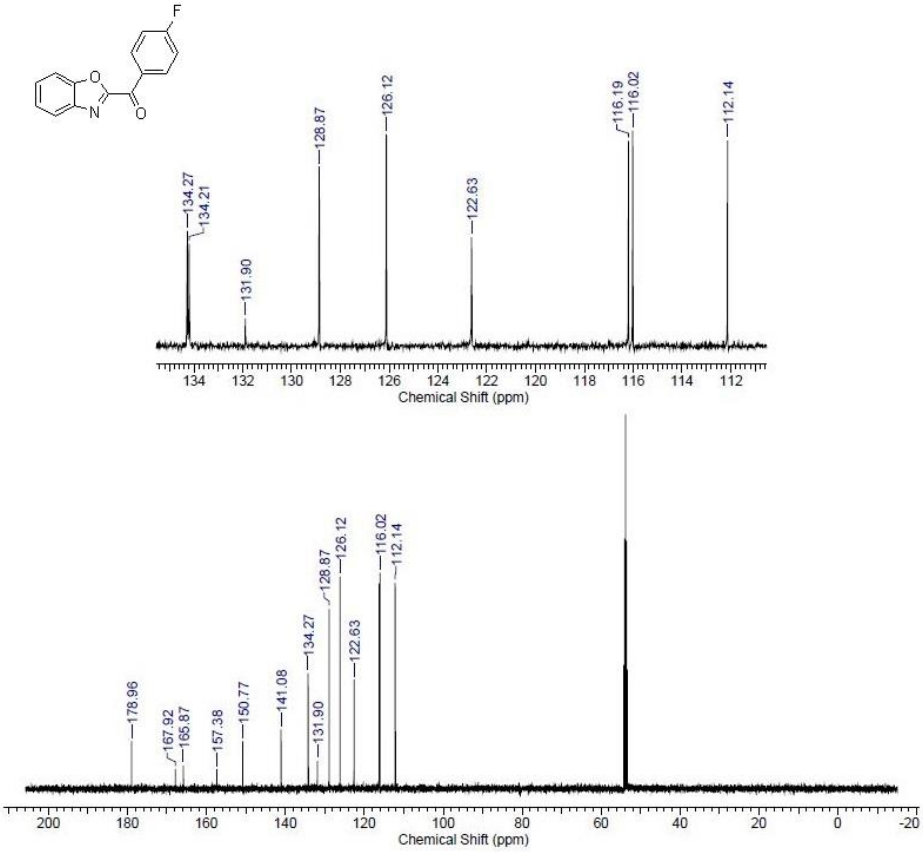

Fig. 19. ^{1}H NMR spectrum ofbenzo[d]oxazol-2-yl(4-methoxyphenyl)methanone (**8c**) (300 MHz, CD_2Cl_2)

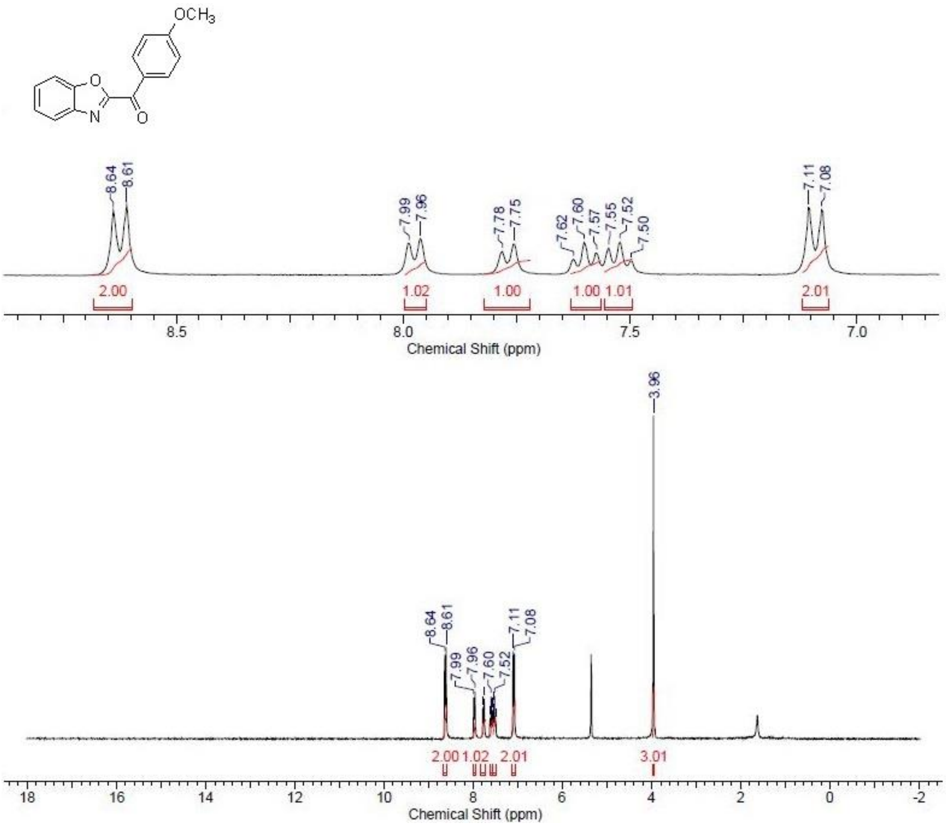

Fig. 20. ^{13}C NMR spectrum of benzo[d]oxazol-2-yl(4-methoxyphenyl)methanone (**8c**) (75 MHz, CD_2Cl_2)

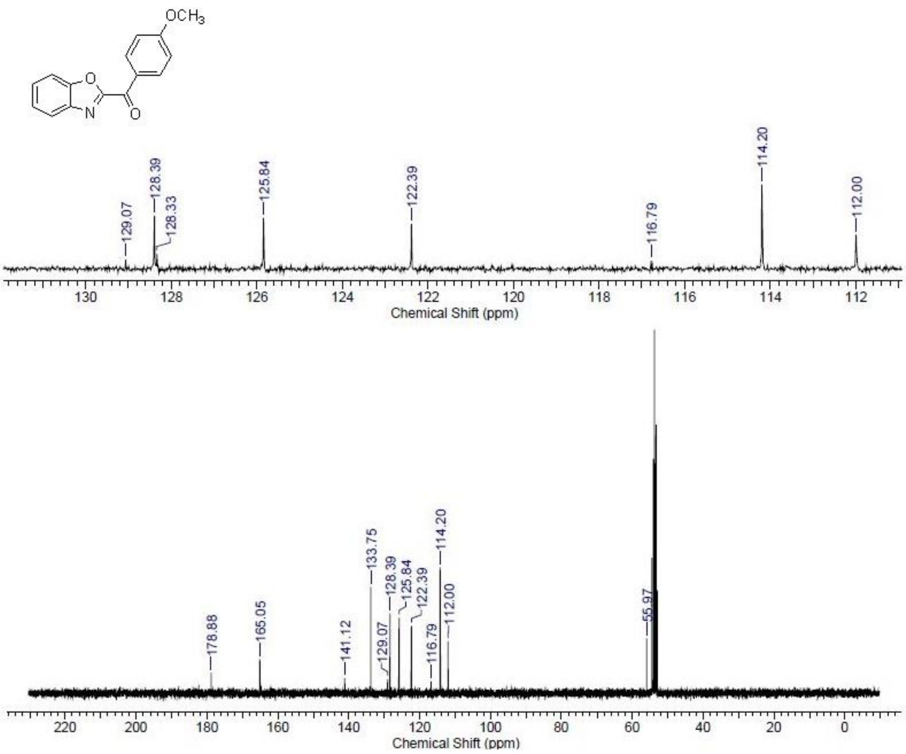

Fig. 21. ^{1}H NMR spectrum of benzo[d]oxazol-2-yl(4-bromophenyl)methanone (**8d**) (300 MHz, CD_2Cl_2)

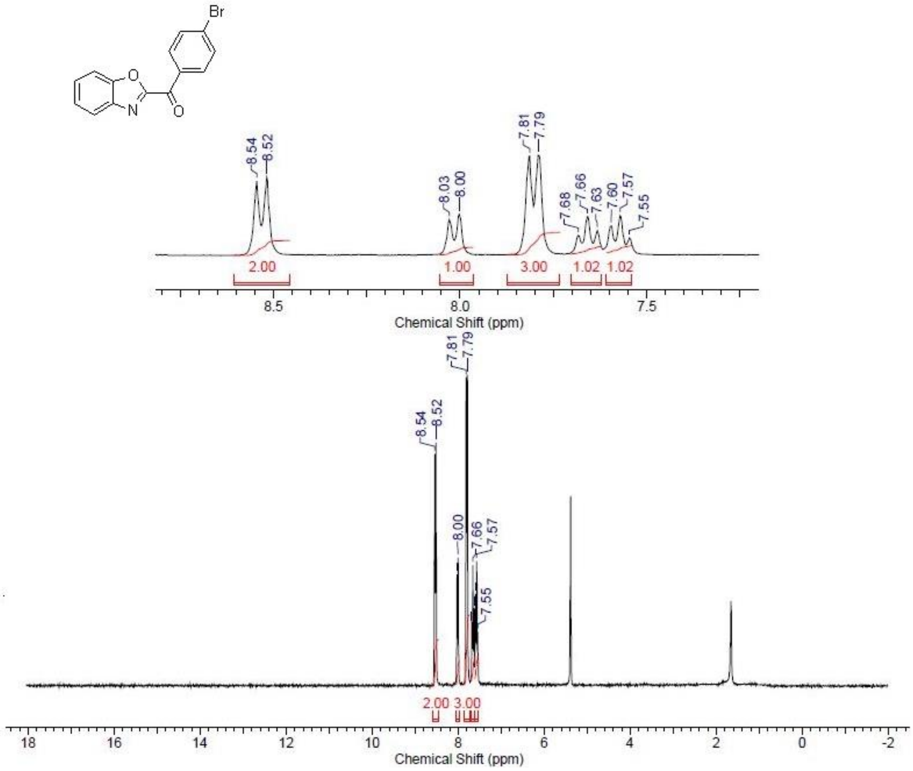

Fig. 22. ^{13}C NMR spectrum of benzo[d]oxazol-2-yl(4-bromophenyl)methanone (**8d**) (125 MHz, CD_2Cl_2)

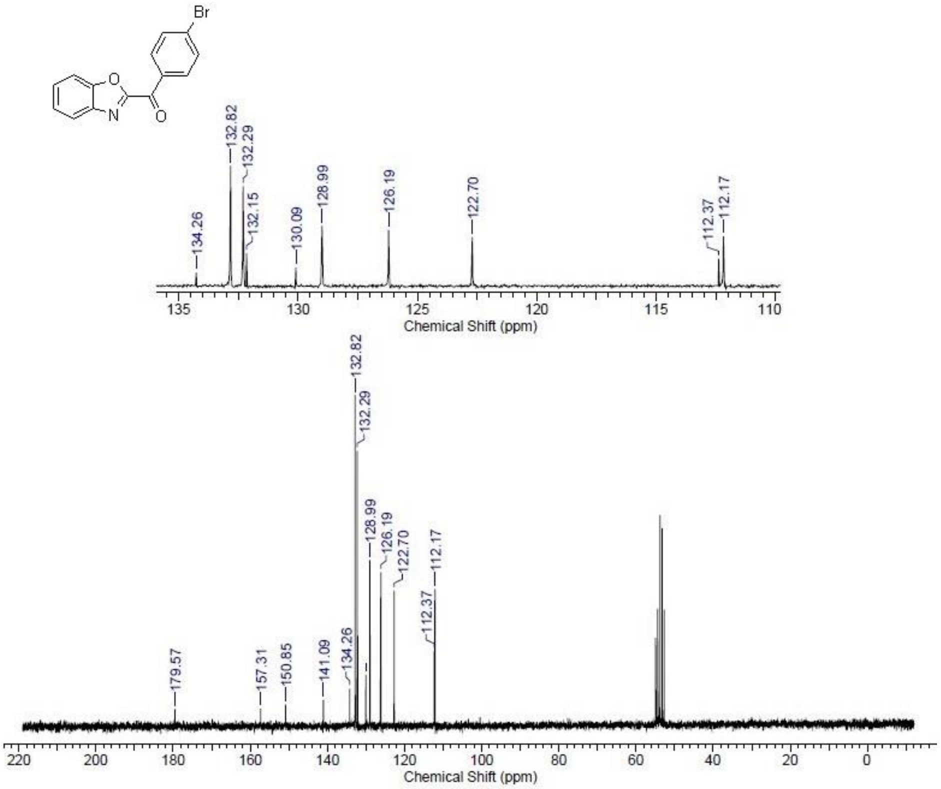

Fig. 23. ^{1}H NMR spectrum of benzo[d]oxazol-2-yl(4-nitrophenyl)methanone (**8e**) (500 MHz, CD_2Cl_2)

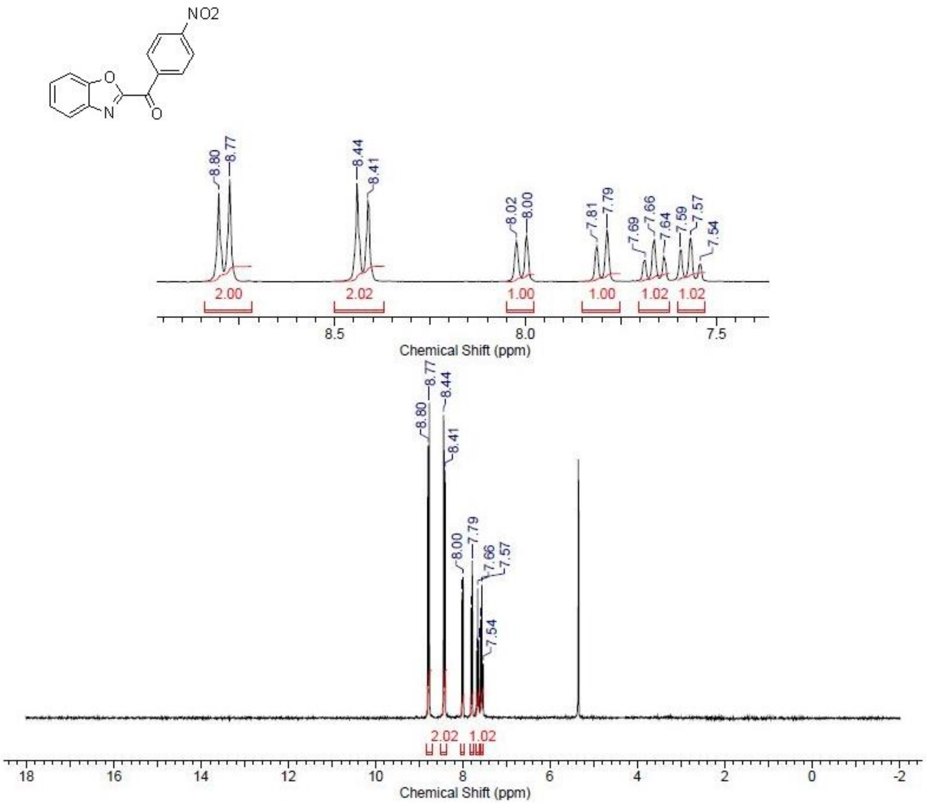

Fig. 24. ^{13}C NMR spectrum of benzo[d]oxazol-2-yl(4-nitrophenyl)methanone (**8e**) (125 MHz, CD_2Cl_2)

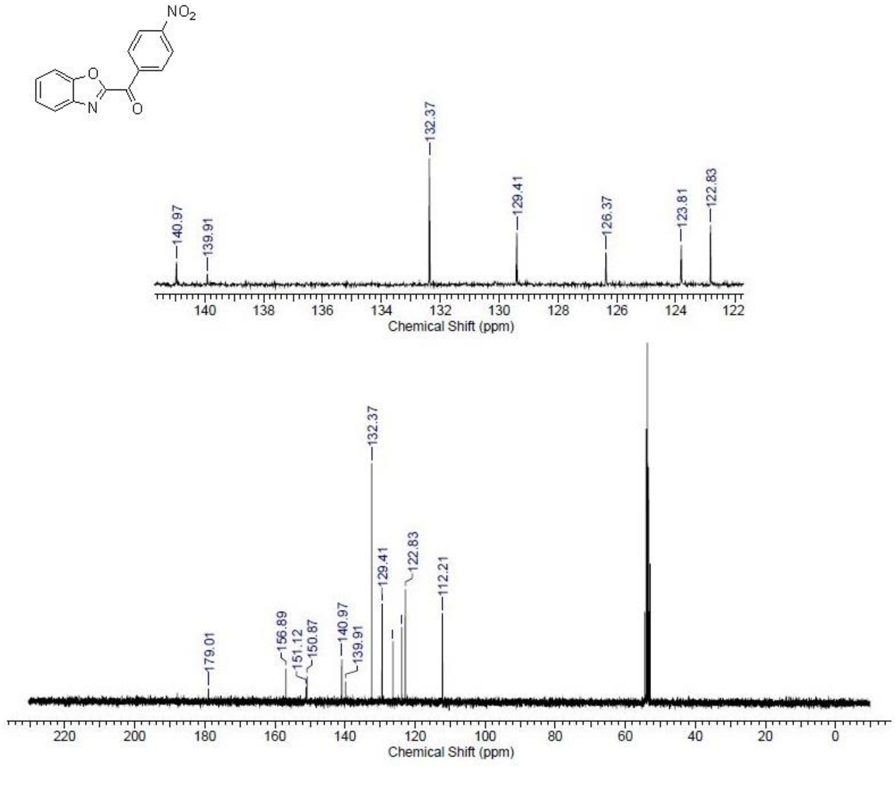

Fig. 25. ^{1}H NMR spectrum of benzo[d]oxazol-2-yl(thiophen-2-yl)methanone (**8f**) (500 MHz, CD_2Cl_2)

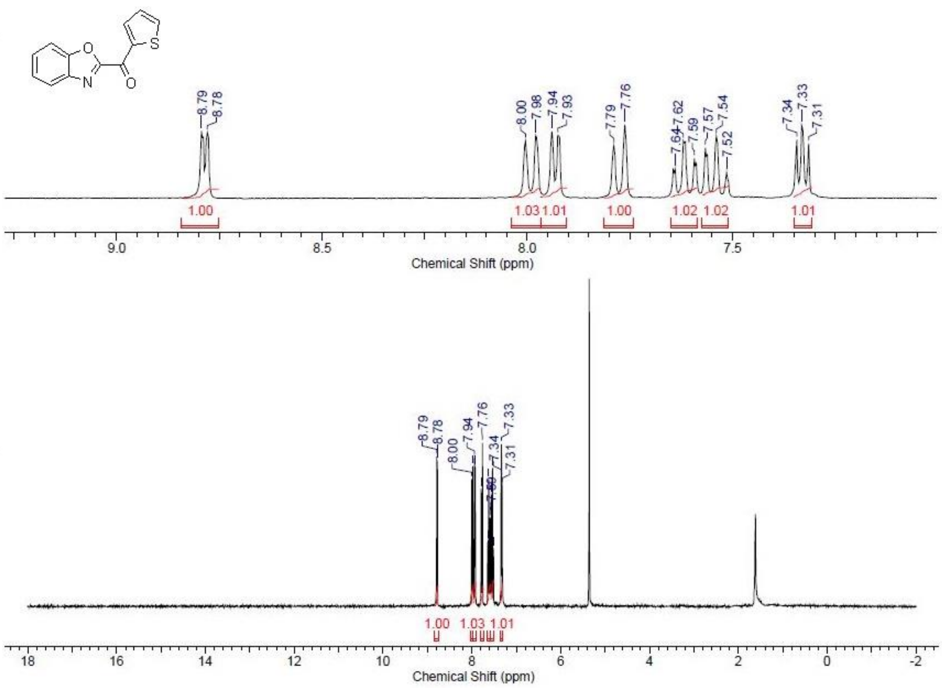

Fig. 26. ^{13}C NMR spectrum of benzo[d]oxazol-2-yl(thiophen-2-yl)methanone (**8f**) (125 MHz, CD_2Cl_2)

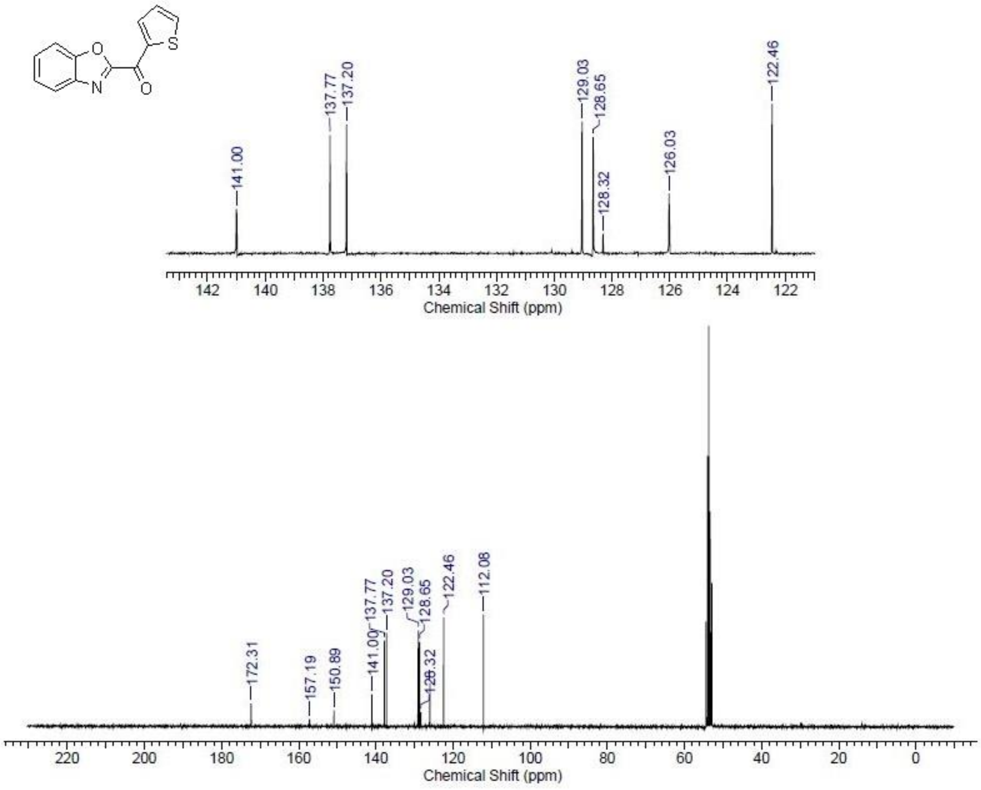

Fig. 27. ^{1}H NMR spectrum of benzo[d]oxazol-2-yl(benzofur-2-yl)methanone (**8g**) (75 MHz, CD_2Cl_2)

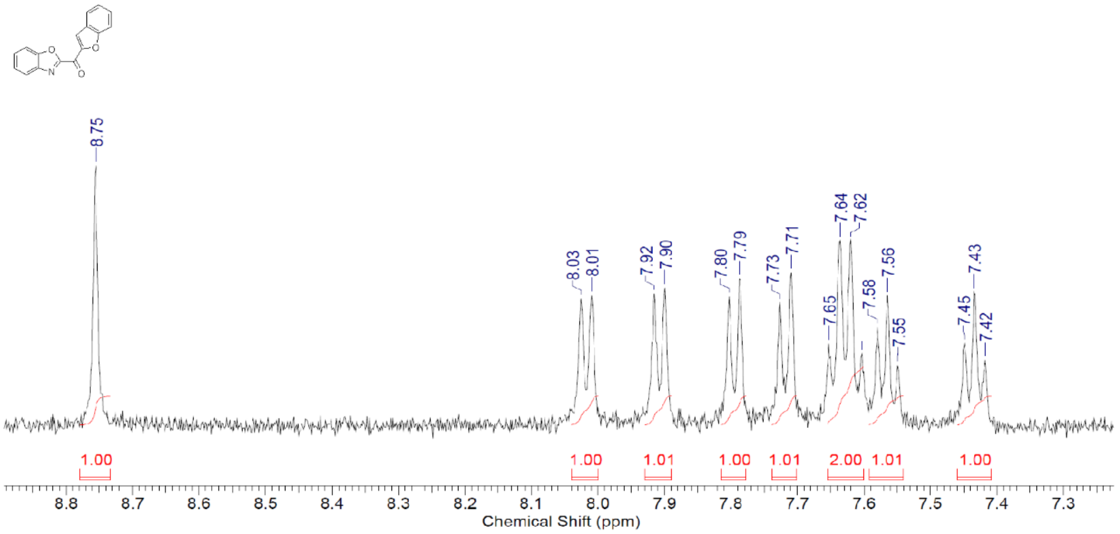

Fig. 28. ^{13}C NMR spectrum of benzo[d]oxazol-2-yl(benzofur-2-yl)methanone (**8g**) (75 MHz, CD_2Cl_2)

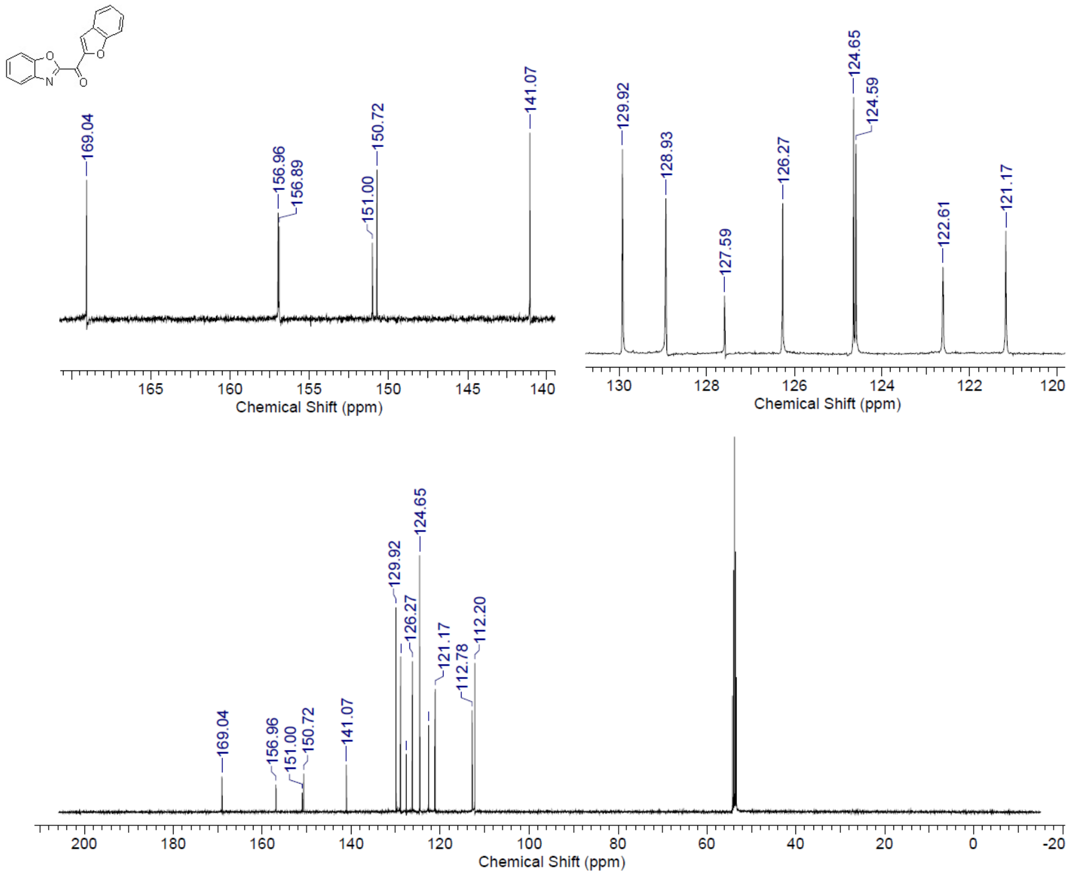

Fig. 29. ^{1}H NMR spectrum of benzo[d]oxazol-2-yl(phenyl)methanimine (**9a**) (300 MHz, CD_3OD)

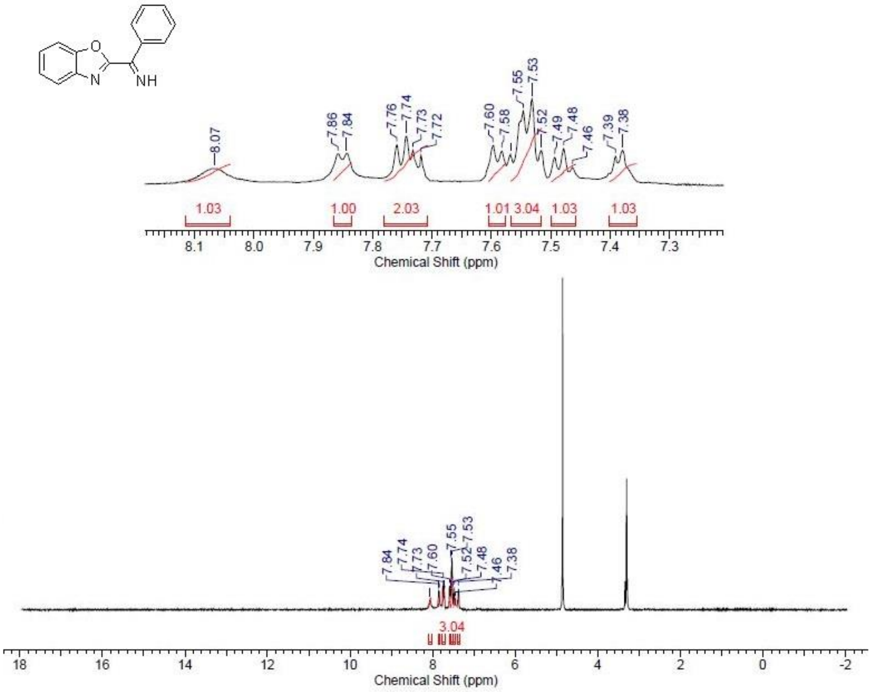

Fig. 30. ^{13}C NMR spectrum of benzo[d]oxazol-2-yl(phenyl)methanimine (**9a**) (300 MHz, CD_3OD)

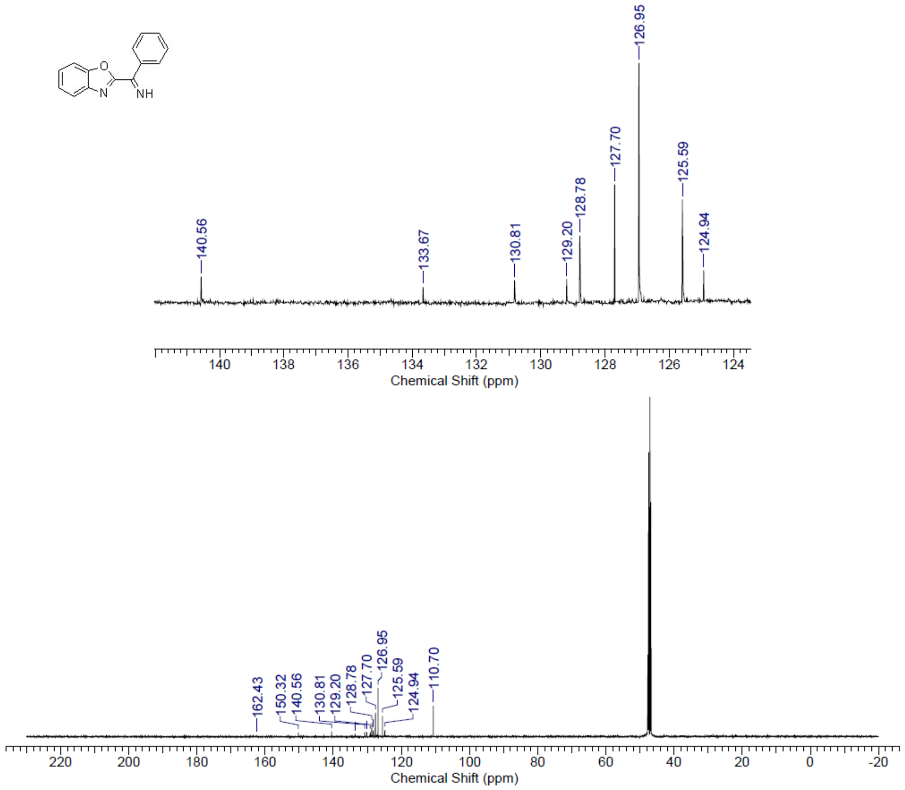

Fig. 31. ^{1}H NMR spectrum of benzo[d]oxazol-2-yl(4-fluorophenyl)methanimine (**9b**) (300 MHz, CD_3OD)

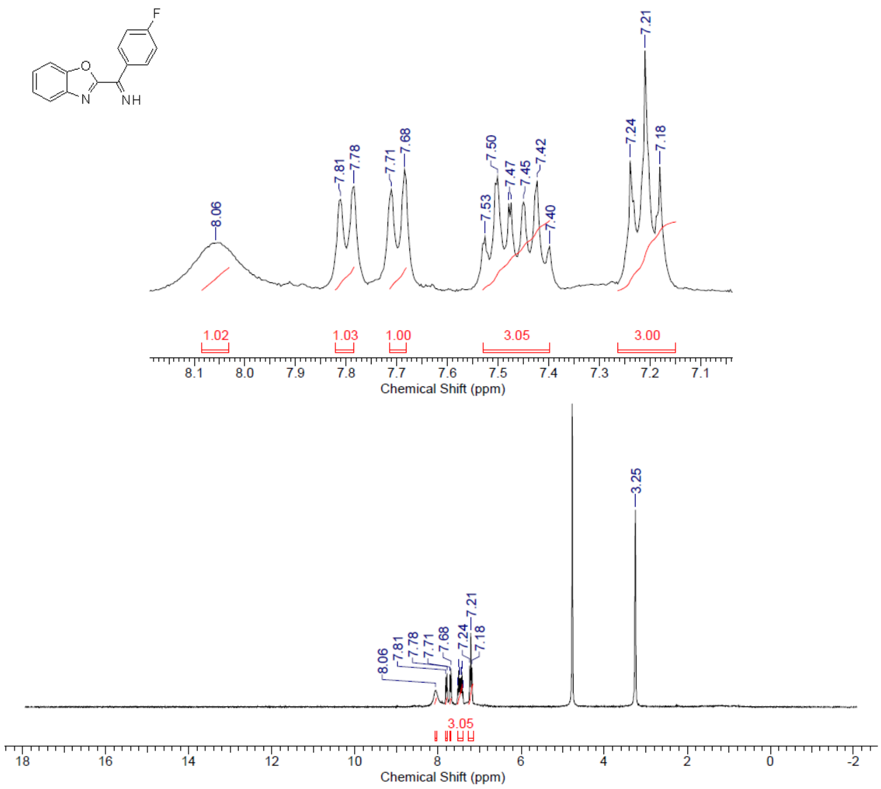

Fig. 32. ^{13}C NMR spectrum of benzo[d]oxazol-2-yl(4-fluorophenyl)methanimine (**9b**) (300 MHz, CD_3OD)

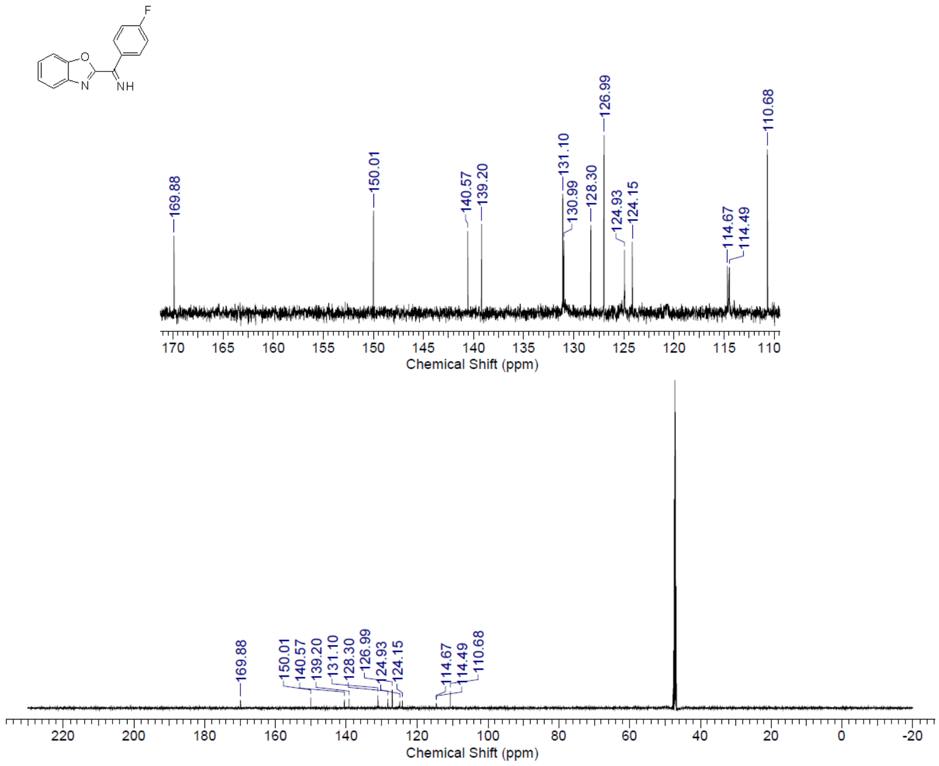

Fig. 33. ^{1}H NMR spectrum of benzo[d]oxazol-2-yl(4-methoxyphenyl)methanimine (**9c**) (300 MHz, CD_3OD)

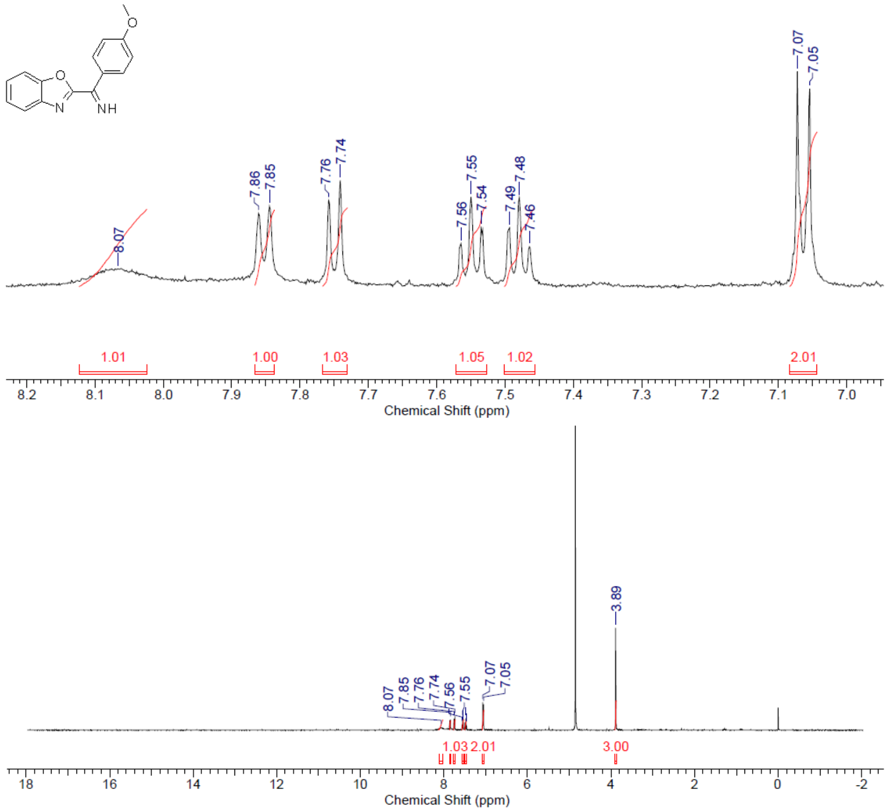

Fig. 34. ^{13}C NMR spectrum of benzo[d]oxazol-2-yl(4-methoxyphenyl)methanimine (**9c**) (300 MHz, CD_3OD)

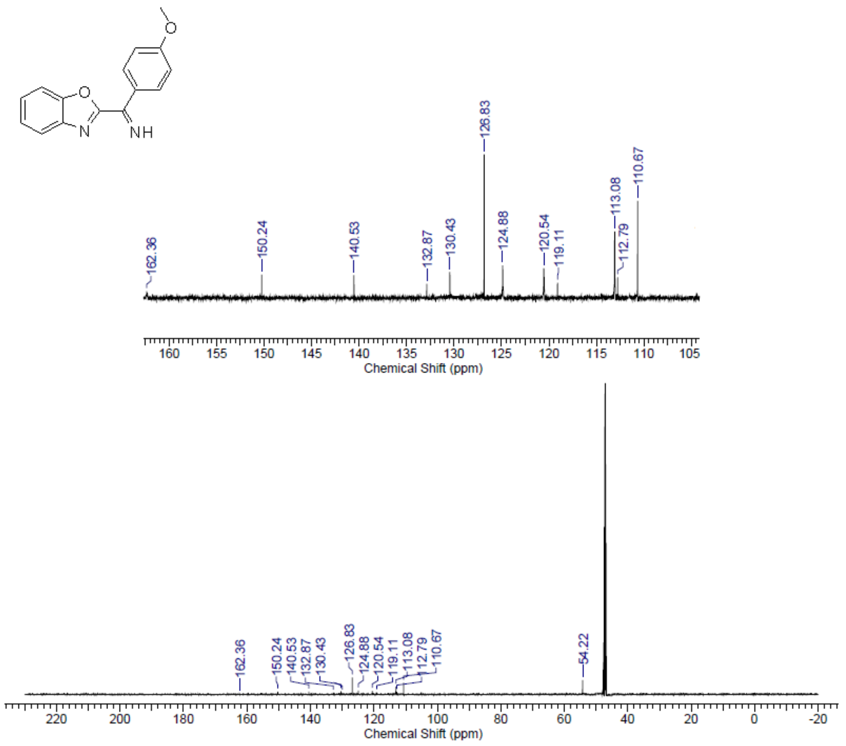

Fig. 35. ^{1}H NMR spectrum of benzo[d]oxazol-2-yl(4-bromophenyl)methanimine (**9d**) (300 MHz, CD_3OD)

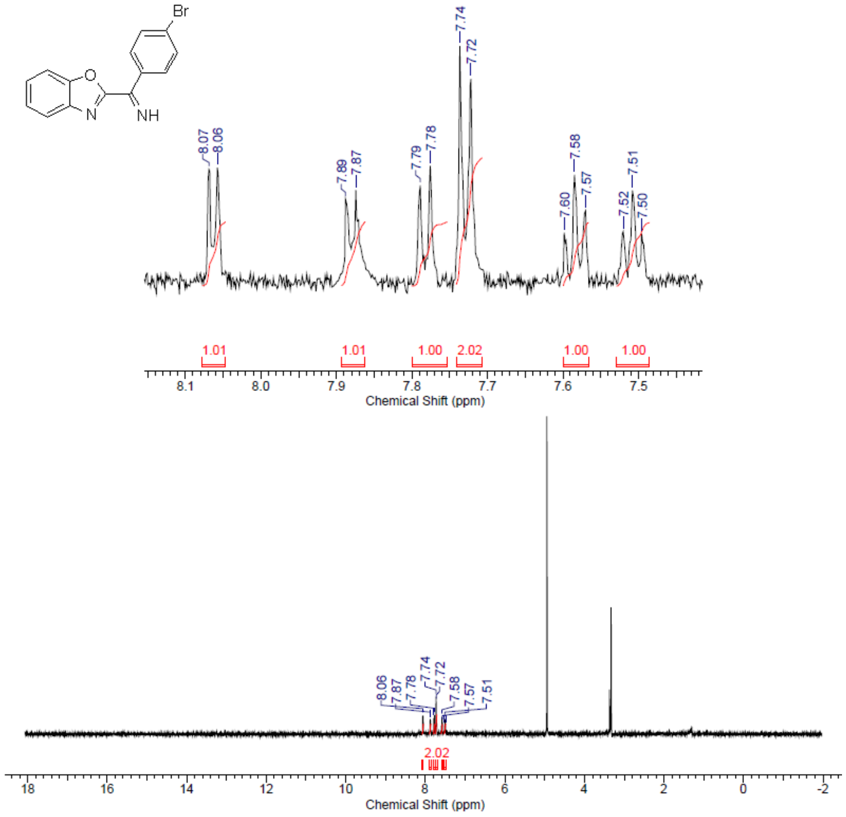

Fig. 36. ^{13}C NMR spectrum of benzo[d]oxazol-2-yl(4-bromophenyl)methanimine (**9d**) (300 MHz, CD_3OD)

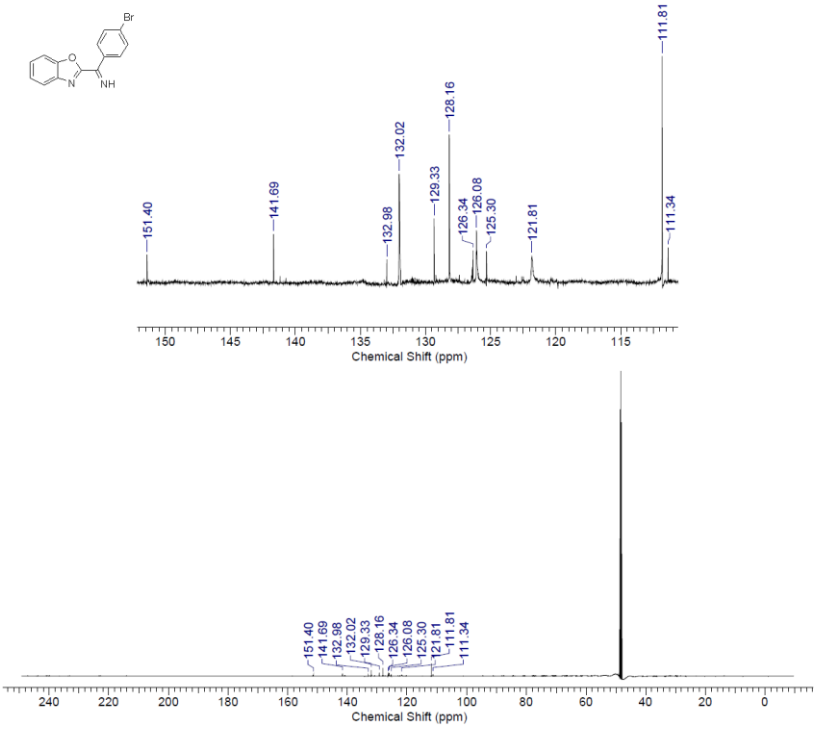

Fig. 37. ^{1}H NMR spectrum of benzo[d]oxazol-2-yl(4-nitrophenyl)methanimine (**9e**) (300 MHz, CD_3OD)

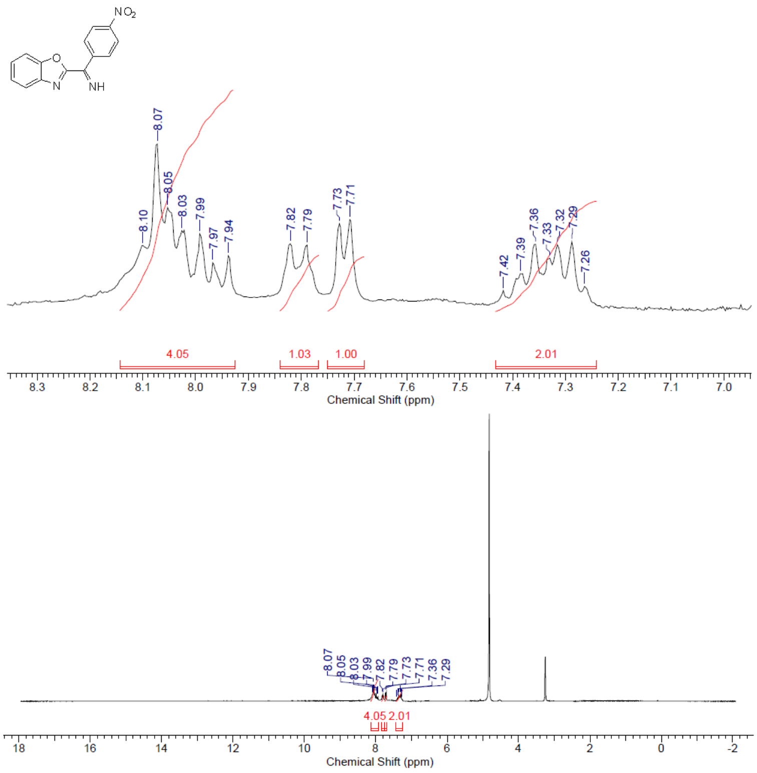

Fig. 38. ^{13}C NMR spectrum of benzo[d]oxazol-2-yl(4-nitrophenyl)methanimine (**9e**) (300 MHz, CD_3OD)

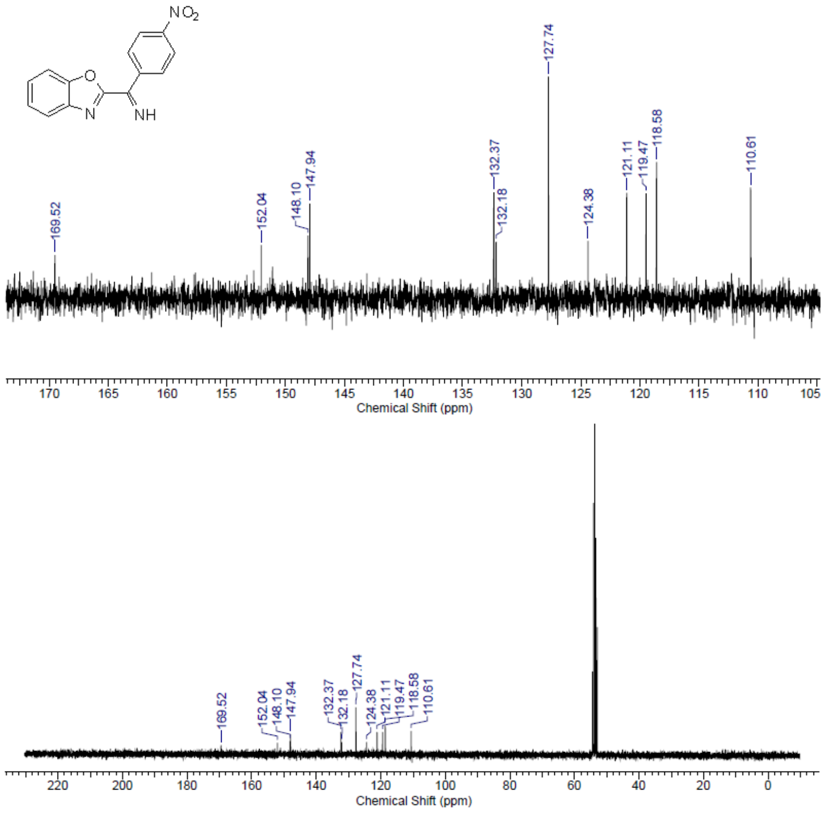

Fig. 39. ^{1}H NMR spectrum of benzo[d]oxazol-2-yl(thiophen-2-yl)methanimine (**9f**) (300 MHz, CD_3OD)

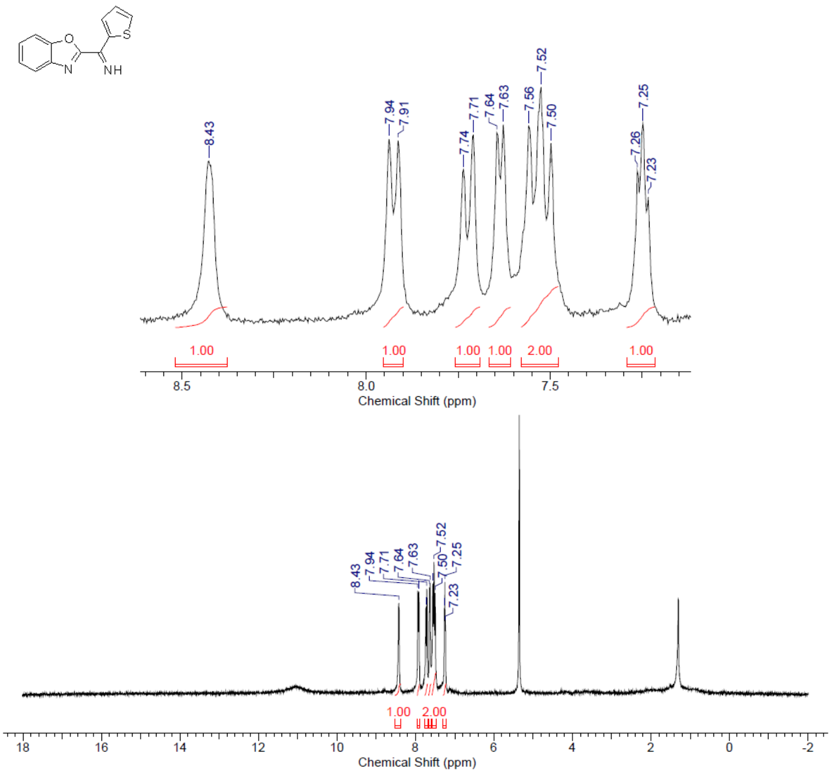

Fig. 40. ^{13}C NMR spectrum of benzo[d]oxazol-2-yl(thien-2-yl)methanimine (**9f**) (300 MHz, CD_3OD)

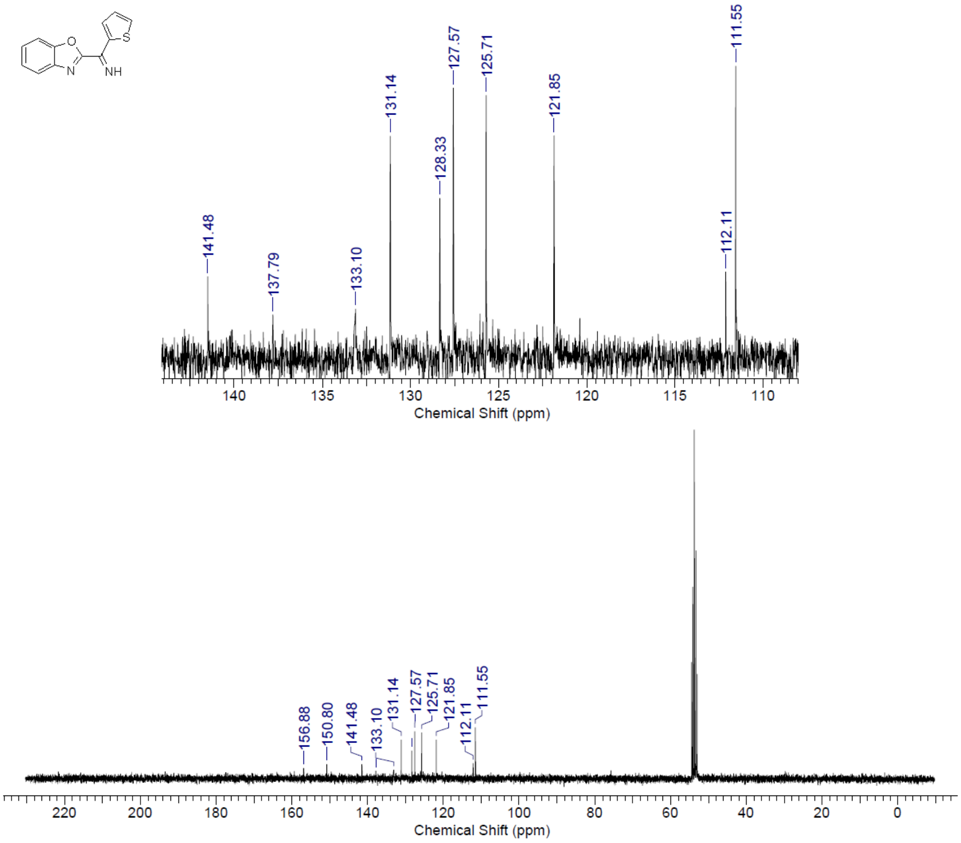

Fig. 41. ^{1}H NMR spectrum of benzo[d]oxazol-2-yl(benzofur-2-yl)methanimine (**9g**) (300 MHz, CD_3OD)

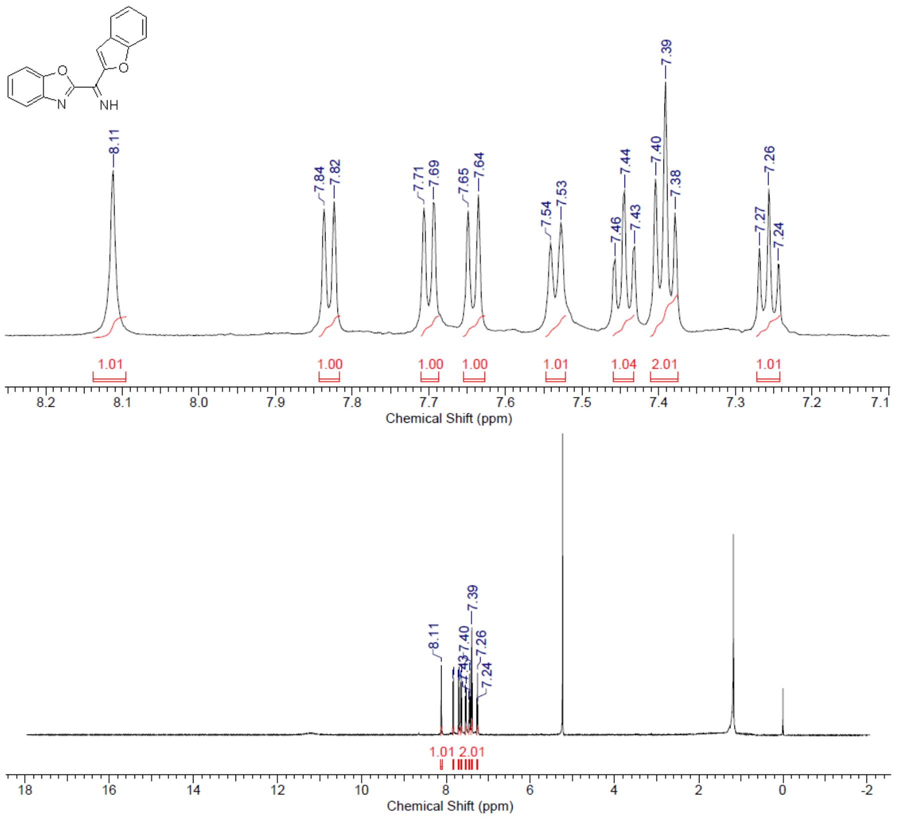

Fig. 42. ^{13}C NMR spectrum of benzo[d]oxazol-2-yl(benzofur-2-yl)methanimine (**9g**) (300 MHz, CD_3OD)

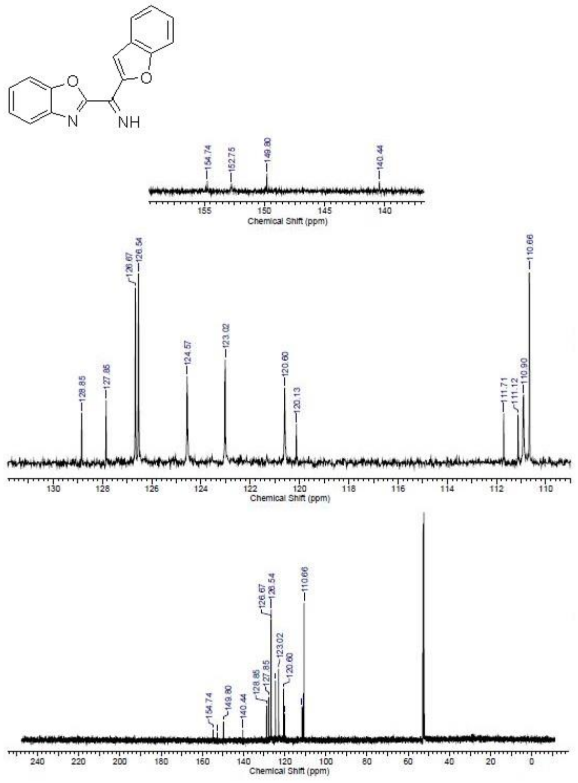

Fig. 43. ^{1}H NMR spectrum of 2-((2-(benzo[d]oxazol-2-yl)-2,5-diphenyl-2H-imidazol-4-yl)amino)phenol (**10a**) (75 MHz, CD_2Cl_2)

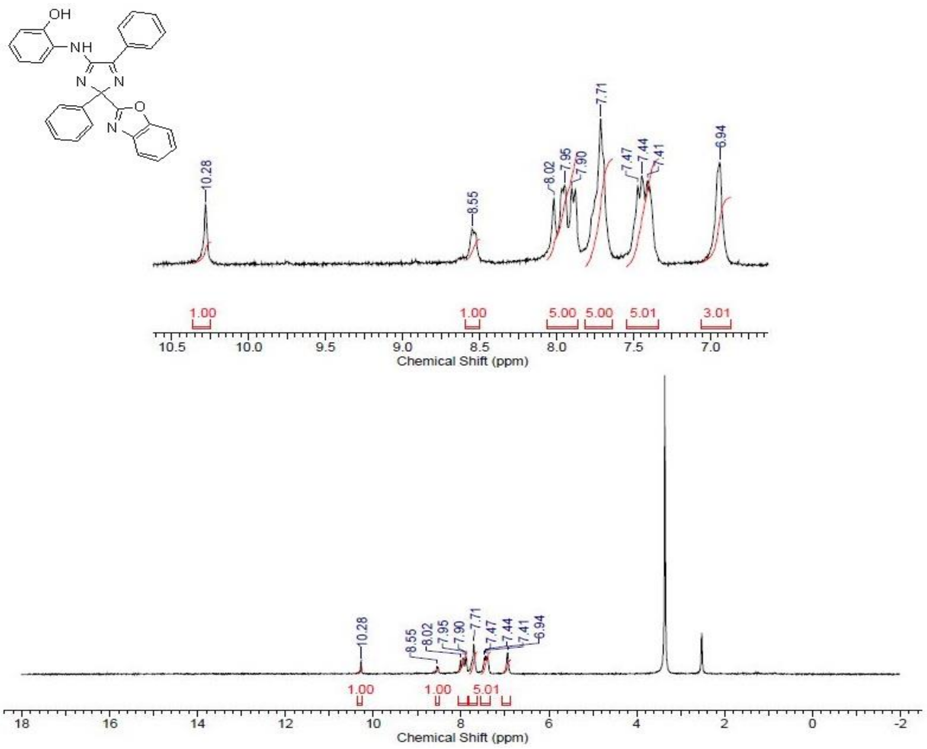

Fig. 44. ^{13}C NMR spectrum of 2-((2-(benzo[d]oxazol-2-yl)-2,5-diphenyl-2H-imidazol-4-yl)amino)phenol (**10a**) (75 MHz, CD_2Cl_2)

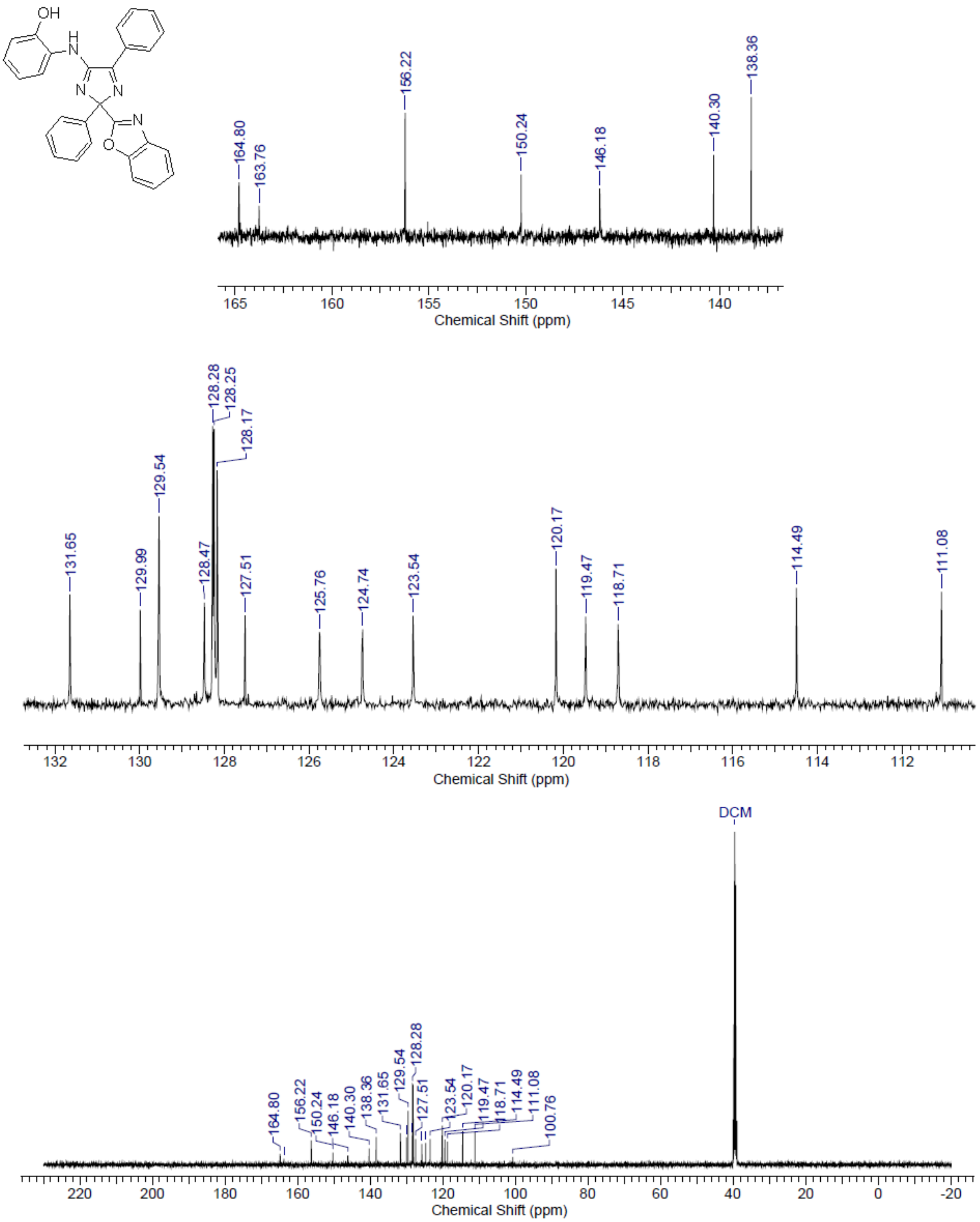

Fig. 45. ^{1}H NMR spectrum of 2-((2-(benzo[d]oxazol-2-yl)-2,5-bis(4-fluorophenyl)-2H-imidazol-4-yl)amino)phenol (**10b**) (300 MHz CD_2Cl_2)

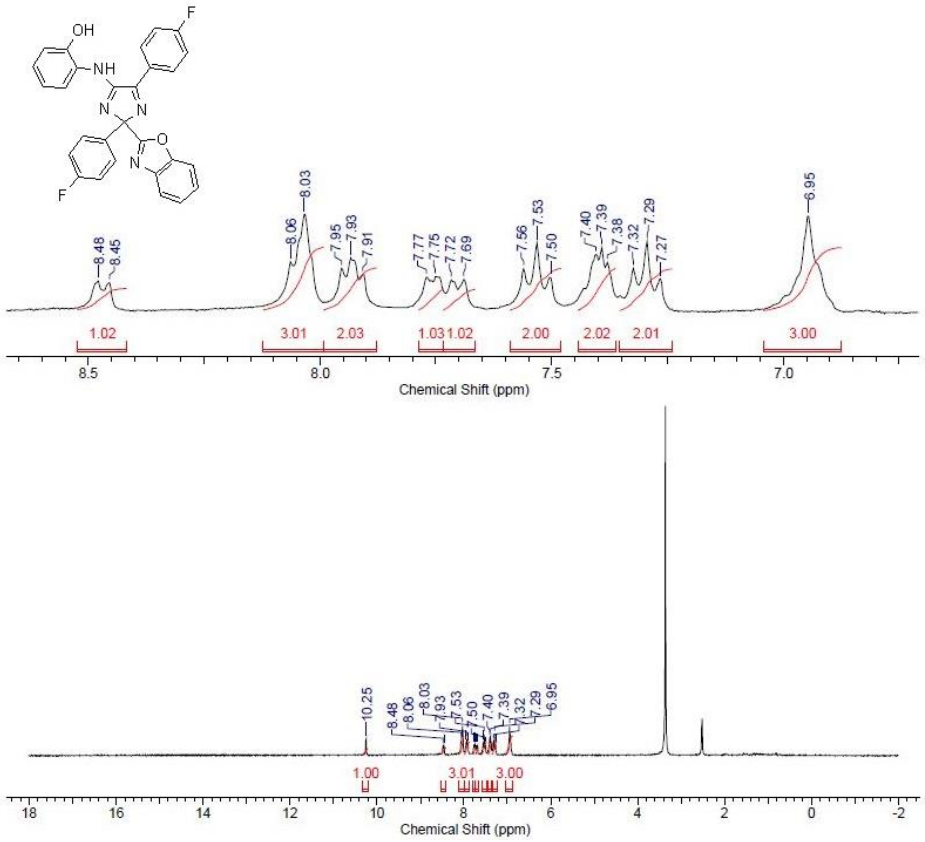

Fig. 46. ^{13}C NMR spectrum of 2-((2-(Benzo[d]oxazol-2-yl)-2,5-bis(4-fluorophenyl)-2H-imidazol-4-yl)amino)phenol (**10b**) (75 MHz, CD_2Cl_2)

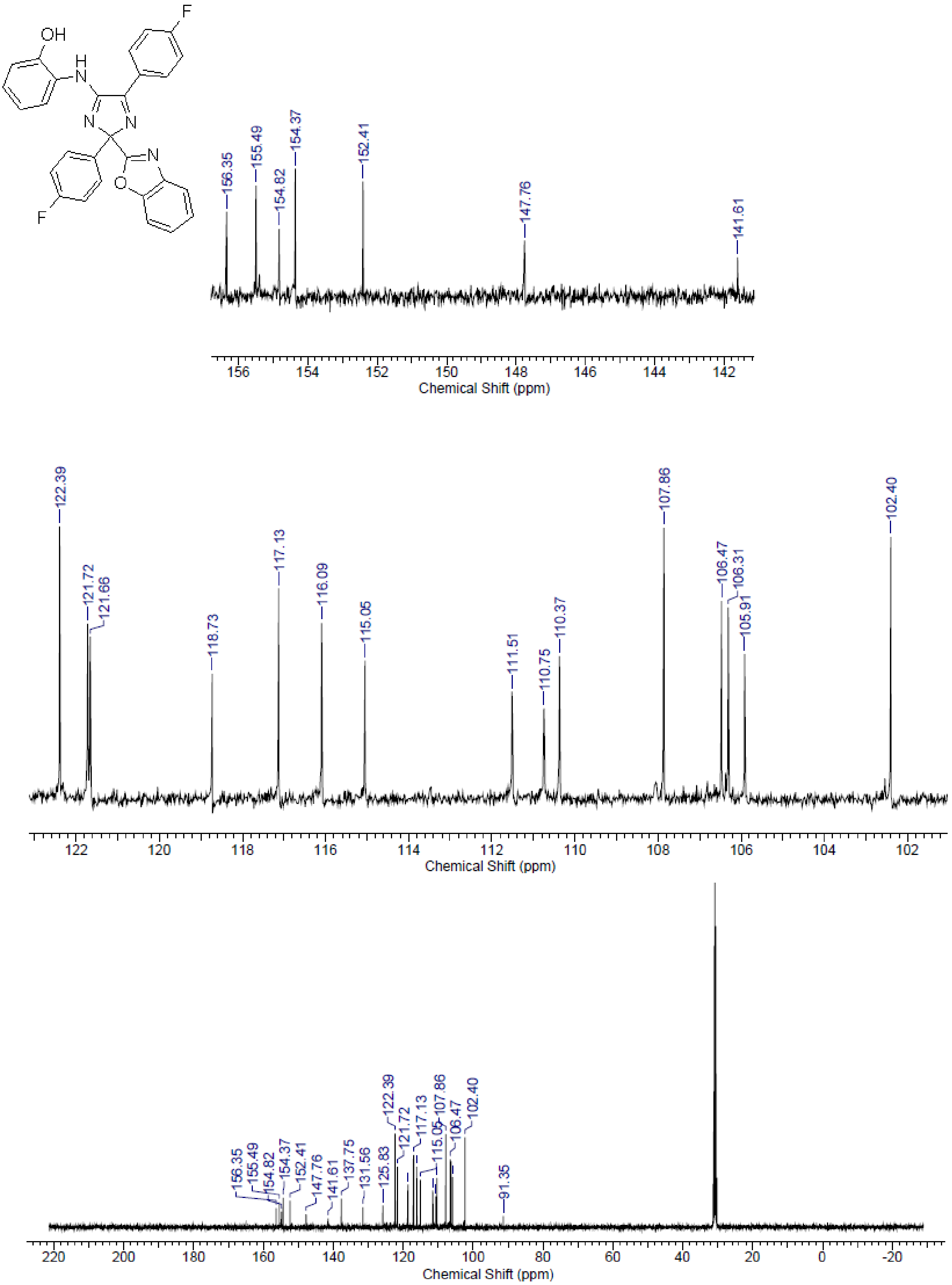

Fig. 47. ^{1}H NMR spectrum of 2-((2-(benzo[d]oxazol-2-yl)-2,5-bis(4-methoxyphenyl)-2H-imidazol-4-yl)amino)phenol (**10c**) (300 MHz, CD_2Cl_2)

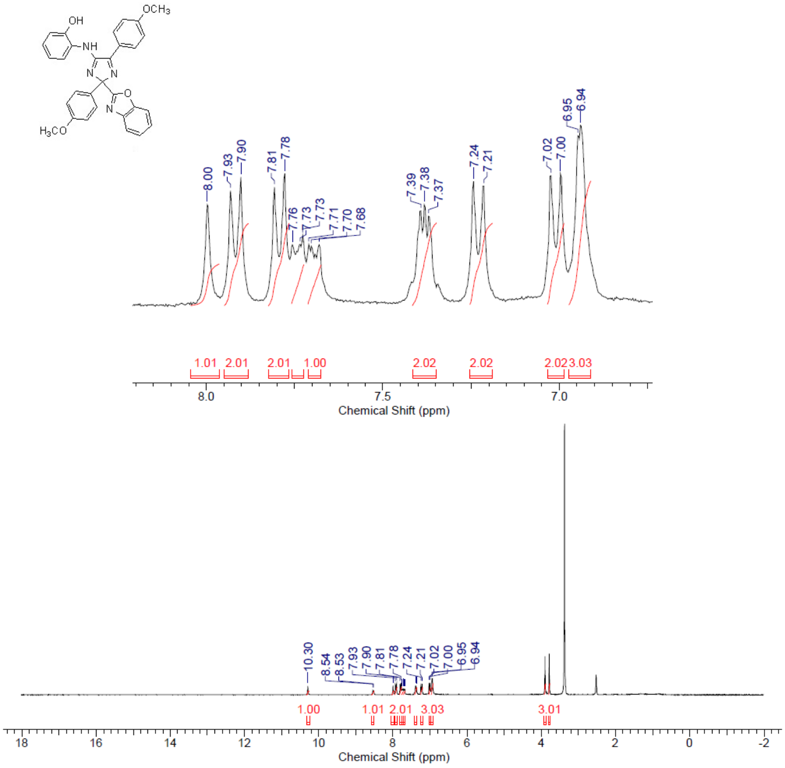

Fig. 48. ^{13}C NMR spectrum of 2-((2-(benzo[d]oxazol-2-yl)-2,5-bis(4-methoxyphenyl)-2H-imidazol-4-yl)amino)phenol (**10c**) (150 MHz, CD_2Cl_2)

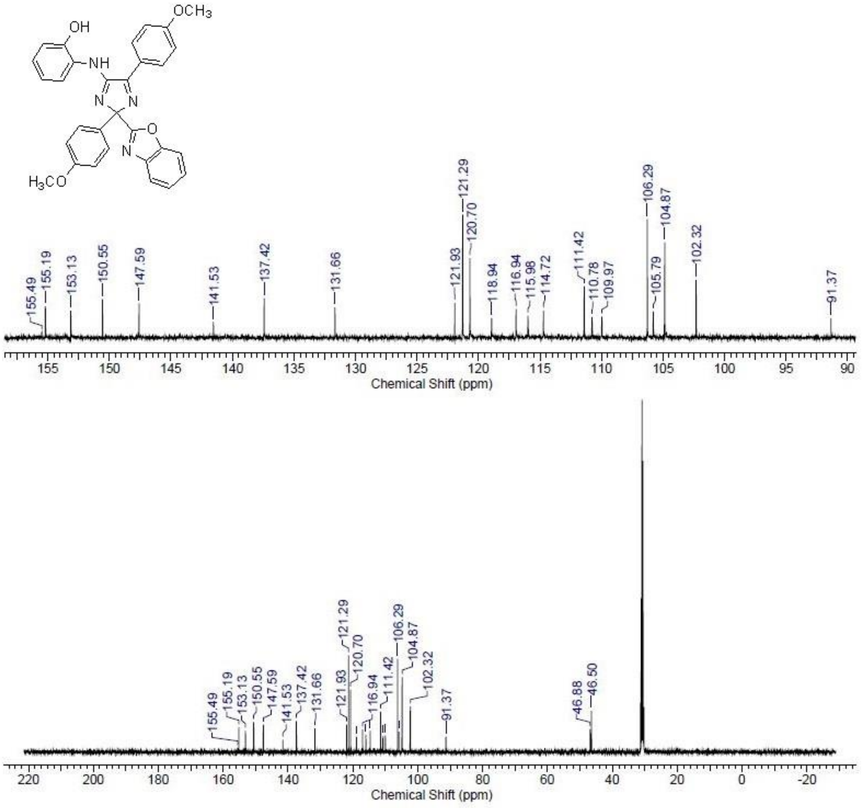

Fig. 49. ^{1}H NMR spectrum of 2-((2-(benzo[d]oxazol-2-yl)-2,5-bis(4-bromophenyl)-2H-imidazol-4-yl)amino)phenol (**10d**) (150 MHz, CD_2Cl_2)

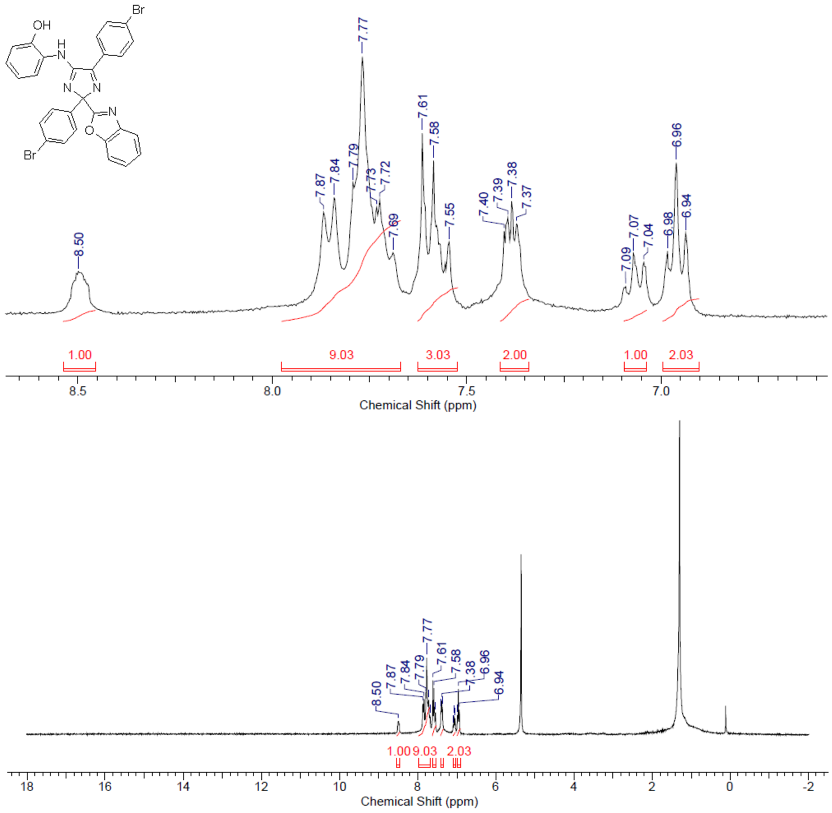

Fig. 50. ^{13}C NMR spectrum of 2-((2-(benzo[d]oxazol-2-yl)-2,5-bis(4-bromophenyl)-2H-imidazol-4-yl)amino)phenol (**10d**) (150 MHz, CD_2Cl_2)

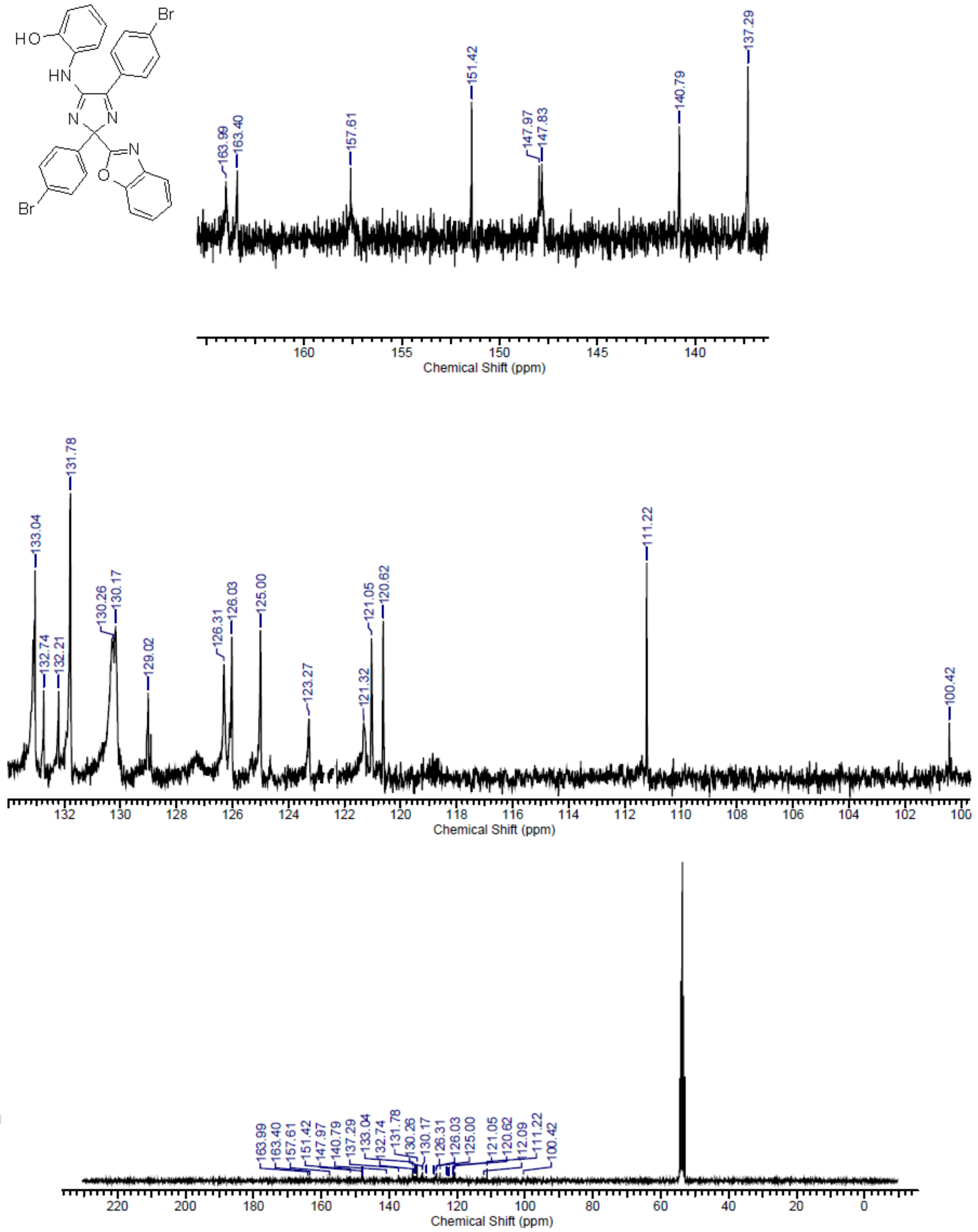

Fig. 51. ^{1}H NMR spectrum of 2-((2-(benzo[d]oxazol-2-yl)-2,5-bis(4-nitrophenyl)-2H-imidazol-4-yl)amino)phenol (**10e**) (300 MHz, CD_2Cl_2)

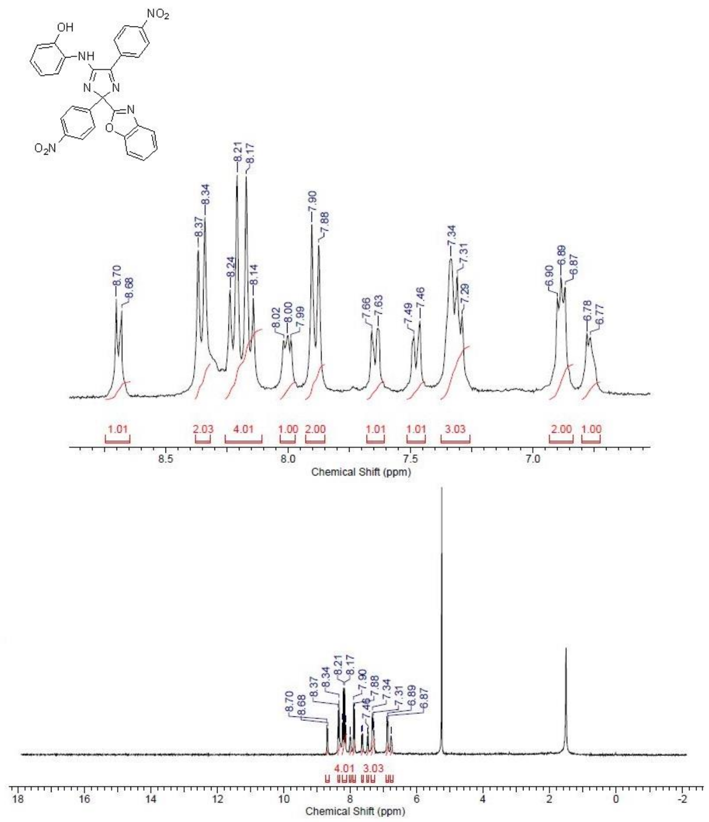

Fig. 52. ^{13}C NMR spectrum of 2-((2-(benzo[d]oxazol-2-yl)-2,5-bis(4-nitrophenyl)-2H-imidazol-4-yl)amino)phenol (**10e**) (150 MHz, CD_2Cl_2)

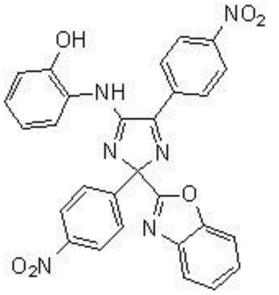

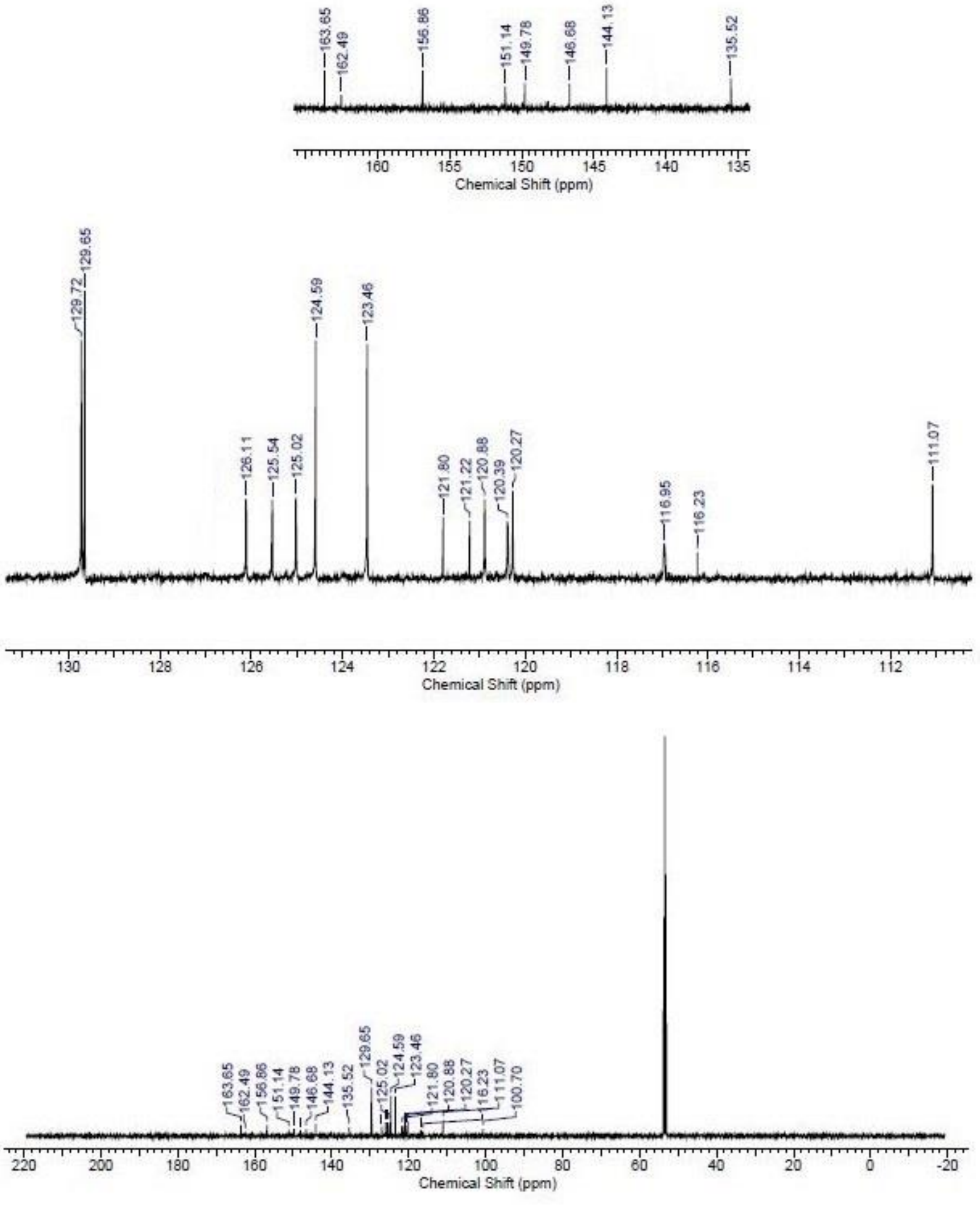

Fig. 53. ^{1}H NMR spectrum of 2-((2-(benzo[d]oxazol-2-yl)-2,5-bis(thiophen-2-yl)-2H-imidazol-4-yl)amino)phenol (**10f**) (300 MHz, CD_2Cl_2)

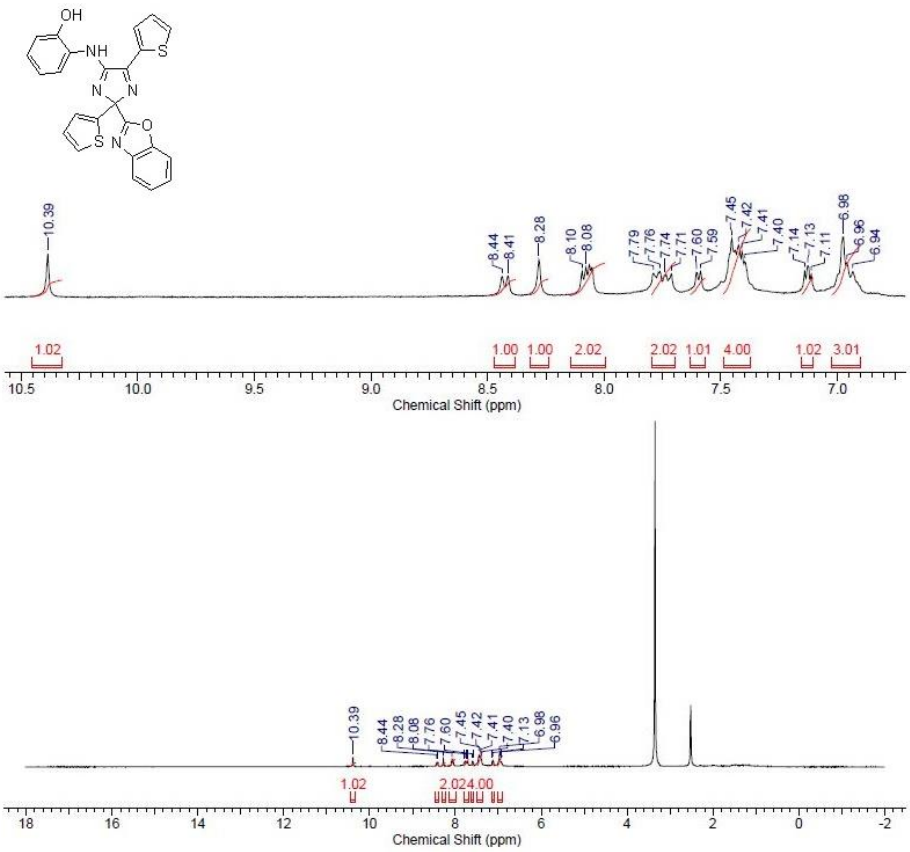

Fig. 54. ^{13}C NMR spectrum of 2-((2-(benzo[d]oxazol-2-yl)-2,5-bis(thiophen-2-yl)-2H-imidazol-4-yl)amino)phenol (**10f**) (150 MHz, CD_2Cl_2)

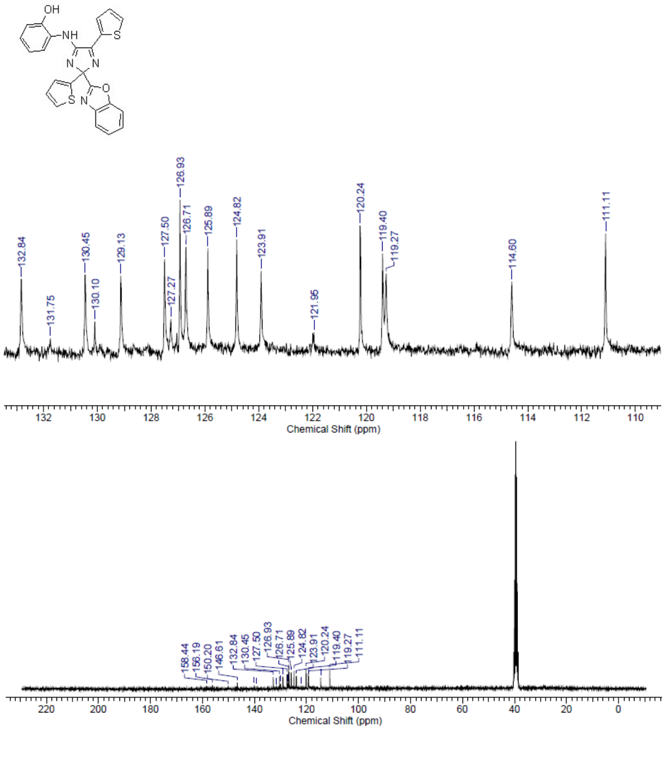

Fig. 55. ^{1}H NMR spectrum of 2-((2-(benzo[d]oxazol-2-yl)-2,5-bis(thiophen-2-yl)-2H-imidazol-4-yl)amino)phenol (**10g**) (300 MHz, CD_2Cl_2)

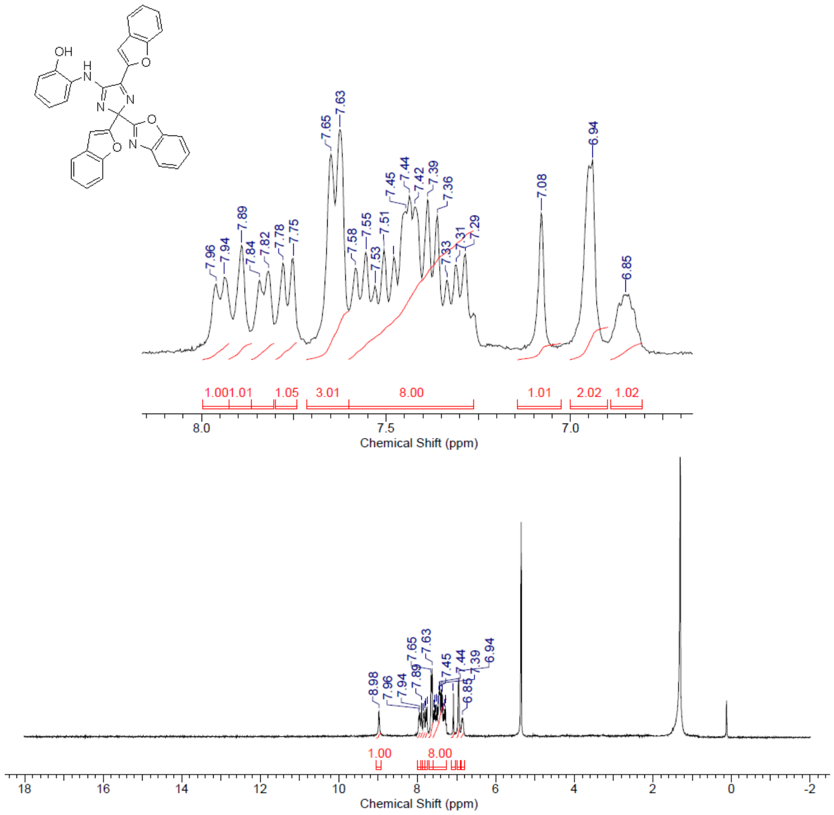

Fig. 56. ^{13}C NMR spectrum of 2-((2-(benzo[d]oxazol-2-yl)-2,5-bis(thiophen-2-yl)-2H-imidazol-4-yl)amino)phenol (**10g**) (150 MHz, CD_2Cl_2)

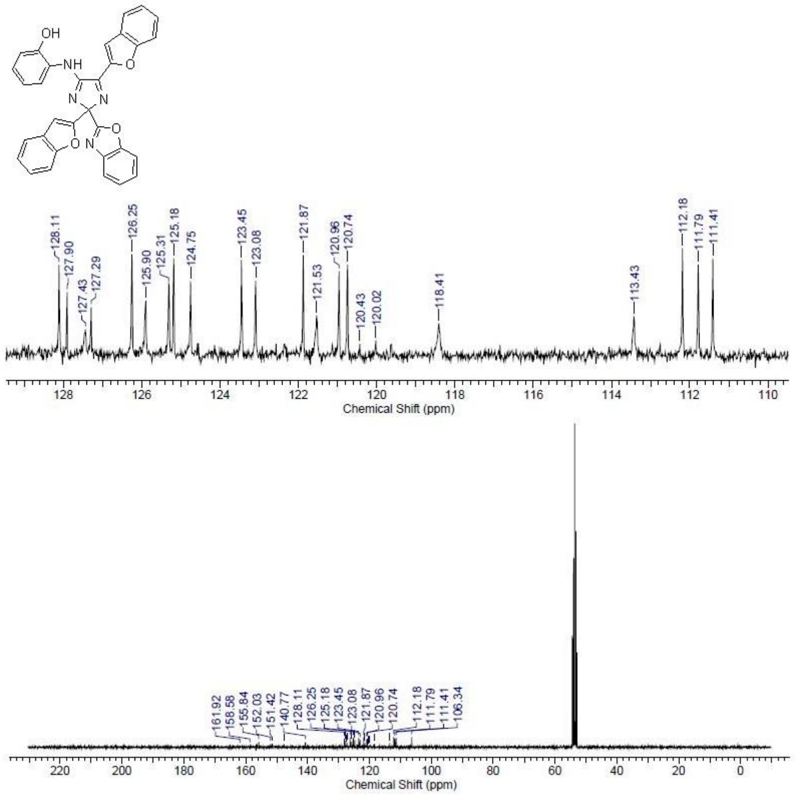

Supplement: Supplementary file 1 [file molecules-25-03768-s001.pdf]
